# Supplementary material for: Planning for Happenstance: Helping Students Optimize Unexpected Career Developments
Source: MedEdPORTAL. 2021 Feb 8;17:11087. doi: 10.15766/mep_2374-8265.11087 (PMC7880249; doi:10.15766/mep_2374-8265.11087)
Supplement: Supplementary file 1 — Eight Stories.docInstructor Guide.docPowerPoint Slides.pptxJason's Story PHLT Video.mp4PHLT Worksheet.docxPHLT Workshop Evaluation.docx [file mep_2374-8265.11087-s001.zip › _Educational_Summary_Report_11087.pdf]

MedEdPORTAL® The AAMC Journal of Teaching and Learning Resources

Original Publication Open Access

# Planning for Happenstance: Helping Students Optimize Unexpected Career Developments

Michelle Vo, MD, Gary Beck Dallaghan, PhD, Nicole Borges, PhD, Anne C. Gill, DrPH, Brian Good, MD, Nathan Gollehon, MD, MHPTT, Jay J. Mehta, MD, MS, Boyd Richards, PhD*, Rachel Richards, MSW, Erna Serelzic, Rebecca Tenney-Soeiro, MD, MSEd, Jason Winward, MD, Dorene Balmer, PhD

*Corresponding author: boyd.richards@hsc.utah.edu

## Abstract

**Introduction:** Planning for and responding to happenstance is an important but rarely discussed part of the professional development of medical students. We noted this gap while conducting a study of career inflection points of 24 physicians who frequently mentioned how luck had shaped their unfolding careers. A review of the career counseling literature led us to a body of work known as Planned Happenstance Learning Theory (PHLT). PHLT focuses on the attitudes and skills to make happenstance a positive force in one's life. We found no reference to this work in the medical education literature and resolved to address this gap. **Methods:** We created resources for an interactive, 90-minute faculty development workshop. In the workshop, the facilitator used a PowerPoint presentation, vignettes of happenstance, a student testimonial, and a reflection worksheet. We presented and formally evaluated the workshop at three national meetings for health science educators. **Results:** Workshop participants, mostly faculty ($N = 45$), consistently expressed positive regard for the workshop content, organization, and instructional methods, especially the opportunity for guided reflection. A retrospective pre/postevaluation revealed a meaningful increase in knowledge about PHLT attitudes and skills, as well as a commitment to use these skills in promoting professional development. **Discussion:** The skills and attitudes of PHLT are relevant to students' career development. A workshop designed to introduce PHLT skills and attitudes to faculty advisors and mentors can help prepare faculty to promote students' awareness and use of these attitudes and skills.

### Keywords
Happenstance, Luck, Career Development, Faculty Development

## Educational Objectives

By the end of this activity, learners will be able to:

1. Recognize the importance of Planned Happenstance Learning Theory (PHLT) attitudes in career development.
2. Recognize the importance of planned happenstance skills in career development.
3. Express confidence in advising/coaching students and/or peers in use of PHLT attitudes and skills in learners' career development.

**Citation:**
Vo M, Beck Dallaghan G, Borges N, et al. Planning for happenstance: helping students optimize unexpected career developments. *MedEdPORTAL*. 2021;17:11087.
https://doi.org/10.15766/mep_2374-8265.11087

## Introduction

Unplanned events can have a dramatic impact on career development.[1,2] Consequently, planning for and responding to happenstance is important but rarely included in discussions of the professional development of medical students. Our study of the career inflection points of 24 physicians who frequently mentioned how luck or happenstance shaped their unfolding career elucidated this gap. We, a team of medical educators at four institutions, received funding from the AAMC's Group on Educational Affairs to explore career inflection points (i.e., influential moments in career trajectories) via in-depth interviews with 24 pediatric faculty at our institutions. In the context of this research, many faculty attributed major career decisions to luck, chance, or serendipity. In contrast, many trainees and students we engage with in educational practice often express discomfort with unplanned events and the uncertainty evoked by unforeseen events.

Copyright © 2021 Vo et al. This is an open-access publication distributed under the terms of the Creative Commons Attribution license.

To explore this gap, we searched the literature and discovered a body of work in vocational counseling known as Planned Happenstance Learning Theory (PHLT).[3-7] Krumboltz theorized that the *journey* of professional development is a function of countless planned and unplanned learning experiences. According to the tenets of PHLT, mentors should work with students guided by several core attitudes. First, they should help students recognize that they can anticipate unplanned events and transform these unplanned events into opportunities for learning. Second, they should help students embrace open-mindedness and consider this openness as tolerance for ambiguity instead of as indecision. Therefore, through PHLT and PHLT-informed mentorship, uncertainty (and the anxiety and distress associated with unplanned events) is reframed as opportunity. Mentors can further help students optimize their response to chance opportunities by cultivating student abilities in the following PHLT skills:

1. Curiosity: exploring new learning opportunities.
2. Persistence: exerting effort despite setbacks.
3. Flexibility: changing attitudes and circumstances.
4. Optimism: viewing new opportunities as possible and attainable.
5. Risk-taking: taking action in the face of uncertain outcomes.

PHLT is used in career counseling outside of medicine. However, to our knowledge, there is no literature describing its use in medical professional planning or medical trainee mentorship or advising. We also could not locate any curricula about addressing happenstance in *MedEdPORTAL* using search terms such as *happenstance*, *luck*, *serendipity*, and *chance*.

We sought to promote PHLT attitudes and principles in professional development in medical education. To that end, we conducted three exploratory focus groups consisting of undergraduate medical students and medical education faculty, followed by three pilot workshops with students at two of the four partner institutions to evaluate students' receptivity and willingness to employ PHLT attitudes and skills. Students consistently expressed appreciation for the opportunity to learn about PHLT and reflect on its application to their career development. For example, one first-year student commented:

> PHLT gives me the mindset that everything will work out. I don't think it relieves me of my personal efforts or robs me of my agency. But to believe we have total control of our futures is a false notion in my opinion. PHLT helps bring our own dedicated efforts into perspective because no matter how hard or smart we work, life runs its course, which is full of twists and turns. I believe it will help me deal with unexpected outcomes and ease disappointments.

In this *MedEdPORTAL* submission, we describe the resources created for use in faculty development workshops to diffuse PHLT attitudes and skills to medical students for career development.

## Methods

### Design of Workshop Resources

We used faculty interviews of career inflection points as a starting point for workshop resources. We selected two interviews from each school ($N = 8$), from which we extracted qualitative data related to PHLT. We used these extracted data to compose narrative summaries. Summaries are included in Appendix A. We iteratively designed, piloted, evaluated, and revised resources for this faculty development workshop in PHLT. The following resources were used to conduct the 90-minute workshop:

- Written stories (Appendix A) showcased how eight faculty members responded to happenstance events in their careers, which were derived from the faculty interviews and focused on PHLT concepts and skills. Ben's story was used in the workshop.
- The Instructor Guide (Appendix B) contained instructions for preparing and conducting the workshop. The guide was informed by feedback from workshop participants and includes facilitator notes.
- The PowerPoint slides (Appendix C) contained objectives, the agenda, content summaries, and presenter notes.
- A video testimonial (Appendix D) from a fourth-year medical student highlighted the impact of happenstance in his professional planning and development.
- A participant worksheet (Appendix E) helped solidify understanding of PHLT. The worksheet facilitated a review of happenstance in one or more narrative stories and helped participants to organize personal reflections of happenstance for professional planning.
- A three-part evaluation (Appendix F) was completed by participants at the end of each workshop.

### Workshop Implementation

This 90-minute faculty development workshop on PHLT was created for faculty in the health sciences who mentor students, either formally or informally, with respect to students' professional planning and development. Participation in the workshop equipped faculty mentors to introduce the concepts, attitudes,

Copyright © 2021 Vo et al. This is an open-access publication distributed under the terms of the Creative Commons Attribution license.

and skills of PHLT to their students. The workshop can be expanded to 120 minutes by allowing more time for each section.

The workshop facilitator was comfortable leading interactive workshops and was familiar with the concepts, attitudes, and skills of PHLT after studying the materials included in the reference list and Appendices A-F. The facilitator completed the reflection worksheet (Appendix E) before the workshop to demonstrate facility with the PHLT concepts and skills. Various facilitators from our interinstitutional team presented the workshop at professional meetings. Before and after each workshop, facilitators from all workshops shared notes and lessons learned.

The workshop was conducted in a medium-sized room with tables that enabled participants to see the projected slides, as well as work both independently and in pairs or small groups.

The facilitator used the Instructor Guide (Appendix B) and PowerPoint slides (Appendix C) to guide the flow of the workshop (Table 1), beginning with introductions and a brief review of the objectives and agenda. After briefly describing the faculty interviews that gave rise to our interest in luck, the facilitator introduced the idea of planning for luck with a quote from Thomas Jefferson and a brief video testimonial from a fourth-year medical student (Appendix D). With participants primed to consider the value of planning for luck, the facilitator introduced the attitudes and skills central to PHLT[3,4] using the PowerPoint slides (Appendix C). Time permitting (workshops lasting more than 75 minutes), the facilitator illustrated application of PHLT attitudes and skills by referencing two movies from pop culture, *Legally Blonde* and *Iron Man*.

In order to transition to participants reflecting on their own use of PHLT attitudes and skills in their professional planning, the facilitator asked participants to read the story of Ben, one of the eight vignettes (Appendix A) captured from our faculty interviews about career inflection points. The facilitator then used the worksheet (Appendix E) to review and discuss how Ben manifested PHLT attitudes and skills. Participants subsequently used the same worksheet to reflect on and make notes about their individual professional trajectory, focusing on the role of unplanned events or happenstance. Participants shared their notes with a peer, using a traditional think-pair-share format. Facilitators circulated among the participants to answer questions, monitor progress, and help participants refocus, if necessary. This activity was followed by a large-group discussion.

**Table 1.** Workshop Time Line (90 Minutes)

| Duration | Content |
|---|---|
| 5 min | Welcome; introductions. |
| 15 min | Background of research. |
| 10 min | Stories and discussion. |
| 10 min | Introduction to PHLT. |
| 15 min | Learning from Ben's story. |
| 15 min | Self-reflection. |
| 20 min | Wrap up and evaluation. |

Abbreviation: PHLT, Planned Happenstance Learning Theory.

The workshop ended with a guided discussion of ways to introduce PHLT to learners and methods to promote PHLT attitudes and skills when advising and mentoring students about ongoing career development.

In the final 5 minutes of the workshop, facilitators asked participants to anonymously complete a workshop evaluation (Appendix F). The results of the evaluation were used to make improvements in content and organization of the workshop materials. This evaluation consisted of three parts, each corresponding to one of the learning objectives: (1) questions asking participants to indicate their level of agreement with statements about the relevance of PHLT attitudes in career development, both prior to and after the workshop (using a retrospective pre/post approach), (2) questions asking participants to indicate their perception of the importance of PHLT skills in career development, and (3) questions asking participants to indicate some aspect of their practice they are most committed to changing through application of PHLT attitudes and skills. The evaluation also asked for narrative feedback about the content and organization of the workshop.

## Results

Workshop participants ($N = 45$) were mostly faculty who work with students as formal or informal mentors and advisors regarding career development. Approximately six students participated in the workshop at the Southern Group on Educational Affairs and Western Group on Educational Affairs regional meetings.

With regard to achievement of the first workshop educational objective, the retrospective pre/postworkshop evaluation indicated that participants recognized the importance of PHLT attitudes immediately following the workshop ($n = 39$). Participants' postworkshop ratings were consistently higher than their retrospective preworkshop ratings in terms of their level of agreement with recognizing the importance of PHLT attitudes (Table 2). For example, agreement with the statement, "Indecision is not a problem to be fixed, but a planful, open-mindedness to future possibilities," increased 1.1 points on a 5-point Likert scale,

Copyright © 2021 Vo et al. This is an open-access publication distributed under the terms of the Creative Commons Attribution license.

**Table 2.** Average Ratings on Retrospective Pre-/Postworkshop Evaluation[a] ($n = 39$)

| Statement | *M* Retrospective Preworkshop Rating | *M* Postworkshop Rating | *M* Increase in Rating |
|---|---|---|---|
| Indecision is not a problem to be fixed, but a planful, open-mindedness to future possibilities. | 3.5 | 4.6 | 1.1[b] |
| It is normal and desirable for unplanned events to influence interests, attitudes, and preferences. | 4.1 | 4.7 | 0.6[b] |
| It is worth the effort to promote and take advantage of happenstance situations. | 4.1 | 4.7 | 0.6[b] |

[a]Rated on a 5-point Likert scale (1 = *strongly disagree*, 5 = *strongly agree*).
[b]$p < .001$.

from 3.5 to 4.6 ($p < .001$). Similar significant increases occurred for the level of agreement with statements, “It is normal and desirable for unplanned events to influence interests, attitudes, and preferences,” and “It is worth the effort to promote and take advantage of happenstance situations.”

With regard to achievement of the second workshop educational objective, participants consistently affirmed the importance of happenstance in their careers and of planning for these unexpected events. The average postworkshop ratings of the importance of the five PHLT skills on a 5-point Likert scale (1 = *low*, 5 = *high*) ranged from 4.5 for risk-taking to 4.8 for persistence.

With regard to achievement of the third workshop educational objective, participants indicated increased confidence in advising/coaching students and/or peers in the use of PHLT attitudes and skills for career development purposes. Average postworkshop ratings of confidence in using each PHTL skill ranged from 3.9 for risk-taking to 4.3 for optimism on a 5-point Likert scale (1 = *low*, 5 = *high*). When asked to identify which PHLT skill(s) they would like to further develop for their own career development, 84% of participants identified at least one skill, from a low of 23% of participants identifying curiosity to a high of 45% of participants identifying risk-taking as a skill they would like to further develop.

We consistently observed active involvement of participants in the workshop’s concluding discussion about ideas for introducing PHLT to learners. Ideas generated in the faculty development workshops included: (1) presenting a similar workshop to learners, (2) helping learners reflect and capture their own stories during informal one-on-one discussions between learners and faculty mentors, and (3) encouraging learners to think about preparing to respond to future happenstance moments as their training progresses in formal career advising sessions.

In terms of the overall quality of the workshop, participant evaluation responses consistently included statements with positive regard for the workshop content and organization. Participants also provided useful constructive feedback, which we used to refine workshop resources and organization. For example, after the first workshop at the annual meeting of the Council on Medical Student Education in Pediatrics, we reduced the amount of time committed to using pop culture references to illustrate PHLT. This modification protected more time for participant personal reflection and discussion about PHLT attitudes and skills in participants’ own career development—the highest regarded aspects of each workshop.

## Discussion

We developed a workshop for faculty who mentor and advise medical students, with the goal of increasing awareness and understanding of PHLT in medical professional development. Based on participants’ level of engagement during the workshops and on feedback from faculty participants, our workshop provided opportunities to discuss the concepts and skills of PHLT. The workshops were positively received by participants, and participants were able to identify how PHLT concepts can enrich professional development and career mentoring discussions with students. The focus groups we conducted with faculty and students showed that both groups recognized the importance of learning about the attitudes and skills required to plan for happenstance to guide learners’ professional development.

As shown through the following student comment, exposing learners to more of the vignettes created from our faculty interviews could have additional benefit:

> I really like taking the physician interviews that have been studied and applying PHLT theory to them; that was a really interesting activity to put PHLT in context. I also think that it was interesting to have access to those stories because I do not have relationships with physicians at this point that let me ask questions about inflection points in their careers.

Indeed, we have found each vignette to be a unique and powerful example of the importance of expecting and responding

Copyright © 2021 Vo et al. This is an open-access publication distributed under the terms of the Creative Commons Attribution license.

to happenstance as one's career unfolds. Nonetheless, we found that during 60- to 90-minute workshops it is difficult to use more than one story. Based on participant feedback, we recommend that workshop presenters protect time for participants to read at least one other story and fill in the workshop worksheet to reflect on that story. We also recommend at least two facilitators for workshops containing up to 20 participants, and more facilitators for workshops with more participants. We found that many participants were eager to share their own stories of planned happenstance during the large-group discussions. Facilitators should be sensitive to the needs of participants while carefully managing time. We learned that faculty participants appreciated time to discuss how PHLT concepts can be applied in their roles as faculty mentors.

To our knowledge, this PHLT workshop is the first faculty development program designed to introduce the PHLT to faculty who mentor health sciences students regarding their career development. Most career development resources used in medical education, specifically those oriented toward career decision-making, emphasize the use of tools to identify personal strengths and the alignment of those strengths with characteristics of specific disciplines.[8] In contrast, this PHLT workshop acknowledges and reframes the uncertainty associated with happenstance and unplanned events in professional development as an opportunity for growth. The PHLT workshop introduces published resources, such as *Luck is No Accident: Making the Most of Happenstance in Your Life and Career*,[9] to faculty mentors in an interactive and engaging manner so that they can internalize the content and prepare to introduce the content to their students. The workshop provides a framework to address what many educators and mentors implicitly understand—opportunities for growth often arise under circuitous and unpredictable circumstances.

### Limitations

The workshop resources provide an introduction to the concepts, attitudes, and skills of PHLT. We recognize that to truly change behavior, more than a 60- to 90-minute workshop is required. Optimally, follow-up reinforcement of learning opportunities must also be sought. We encourage ongoing longitudinal use of the prepared vignettes. The availability of these vignettes may facilitate longitudinal discussions of PHLT concepts and skills and therefore reinforce learning and internalization of a desired growth mindset in medical professional development. Nevertheless, our evaluation data suggest that the workshop as designed does represent an important first step in increasing awareness of PHLT and the likelihood of enhancing its uptake in medical education.

Regarding future directions of the described PHLT work in medical education, we continue to seek additional ways to disseminate our lessons learned about PHLT and how to help learners apply them in their career development. In doing so, we wish to contribute additional resources to learners to assist in the development of a growth mindset and flexibility in approaching professional development in medical education.

## Appendices

A. Eight Stories.doc
B. Instructor Guide.doc
C. PowerPoint Slides.pptx
D. Jason's Story PHLT Video.mp4
E. PHLT Worksheet.docx
F. PHLT Workshop Evaluation.docx

*All appendices are peer reviewed as integral parts of the Original Publication.*

**Michelle Vo, MD:** Assistant Professor, Department of Psychiatry and Director of Student Wellness, University of Utah School of Medicine

**Gary Beck Dallaghan, PhD:** Associate Professor and Director of Educational Scholarship, Office of Medical Education, University of North Carolina

**Nicole Borges, PhD:** Professor and Department Chair, Department of Medical Education, Geisel School of Medicine at Dartmouth

**Anne C. Gill, DrPH:** Professor and Assistant Dean of Interprofessional Education, Department of Pediatrics, Baylor College of Medicine

**Brian Good, MD:** Associate Professor and Pediatric Clerkship Director, Department of Pediatrics, University of Utah School of Medicine

**Nathan Gollehon, MD, MHPTT:** Associate Professor and Vice Chair for Education, Department of Pediatrics, University of Nebraska Medical Center

**Jay J. Mehta, MD, MS:** Associate Professor, Children's Hospital of Philadelphia, Department of Pediatrics, Perelman School of Medicine, University of Pennsylvania

**Boyd Richards, PhD:** Professor and Director of Educational Scholarship and Research, Department of Pediatrics, University of Utah School of Medicine; ORCID: https://orcid.org/0000-0002-1864-7238

**Rachel Richards, MSW:** Visiting Scholar and Intern, University of Utah School of Medicine

Copyright © 2021 Vo et al. This is an open-access publication distributed under the terms of the Creative Commons Attribution license. 

**Erna Serelzic:** Research Assistant, University of Utah School of Medicine

**Rebecca Tenney-Soeiro, MD, MSEd:** Associate Professor, Department of Pediatrics, Children's Hospital of Philadelphia, Perelman School of Medicine, University of Pennsylvania

**Jason Winward, MD:** First-Year Resident, Department of Internal Medicine, The University of Iowa School of Medicine

**Dorene Balmer, PhD:** Associate Professor, Department of Pediatrics, Children's Hospital of Philadelphia, Perelman School of Medicine, University of Pennsylvania

### Disclosures
None to report.

### Funding/Support
Funding support provided by the Association of American Medical Colleges' Group on Educational Affairs.

### Prior Presentations
Beck-Dallagahn G, Gollehan N, Richards B, Good B, Tenney-Soeiro R, Borges N. When opportunity comes a knockin'! Applying planned happenstance to medical student advising. Presented at: Council on Medical Student Education in Pediatrics; March 21, 2019; St Petersburg, FL.

Richards B, Vo M, Winward J, Serelzic E, Richards R. Personal and contextual factors which shape career inflection points: an exploratory qualitative study at four institutions. Presented at: Western Group on Educational Affairs; March 28-31, 2019; Reno, NV.

Mehta J, Balmer D. Personal and contextual factors which shape career inflection points: an exploratory qualitative study at 4 institutions. Presented at: Northeast Group on Educational Affairs; April 4-6, 2019; Philadelphia, PA.

### Ethical Approval
The University of Utah Institutional Review Board approved this study.

## References

1. Bandura A. The psychology of chance encounters and life paths. *Am Psychol*. 1982;37(7):747-755. https://doi.org/10.1037/0003-066X.37.7.747
2. Hirschi A. The role of chance events in the school-to-work transition: the influence of demographic, personality and career development variables. *J Vocatl Behav*. 2010;77(1):39-49. https://doi.org/10.1016/j.jvb.2010.02.002
3. Krumboltz JD. The happenstance learning theory. *J Career Assess*. 2009;17(2):135-154. https://doi.org/10.1177/1069072708328861
4. Krumboltz JD. Capitalizing on happenstance. *J Employ Couns*. 2011;48(4):156-158. https://doi.org/10.1002/j.2161-1920.2011.tb01101.x
5. Knudsen GH, Lemmergaard J. Strategic serendipity: how one organization planned for and took advantage of unexpected communicative opportunities. *Cult Organ*. 2014;20(5):392-409. https://doi.org/10.1080/14759551.2014.948440
6. Lee JH, Cho S, Lee S, et al. Initial validation of the Planned Happenstance Career Inventory–English version. *Career Dev Q*. 2017;65(4):366-378. https://doi.org/10.1002/cdq.12114
7. Yang N, Yaung H, Noh H, Jang SH, Lee B. The change of planned happenstance skills and its association with career-related variables during school-to-work transition. *Int J Educ Vocat Guid*. 2017;17(1):19-38. https://doi.org/10.1007/s10775-016-9332-z
8. Careers in medicine. Association of American Medical Colleges. Accessed September 28, 2020. https://www.aamc.org/cim/
9. Krumboltz JD, Levin AS. Luck is No Accident: Making the Most of Happenstance in Your Life and Career. 2nd ed. Impact Publishers; 2010.

**Received:** June 22, 2020
**Accepted:** October 20, 2020
**Published:** February 8, 2021

Copyright © 2021 Vo et al. This is an open-access publication distributed under the terms of the Creative Commons Attribution license.
