# Supplementary material for: Planning for Happenstance: Helping Students Optimize Unexpected Career Developments
Source: MedEdPORTAL. 2021 Feb 8;17:11087. doi: 10.15766/mep_2374-8265.11087 (PMC7880249; doi:10.15766/mep_2374-8265.11087)
Supplement: Supplementary file 1 — Eight Stories.docInstructor Guide.docPowerPoint Slides.pptxJason's Story PHLT Video.mp4PHLT Worksheet.docxPHLT Workshop Evaluation.docx [file mep_2374-8265.11087-s001.zip › C. PowerPoint Slides.pptx]

## Slide 1
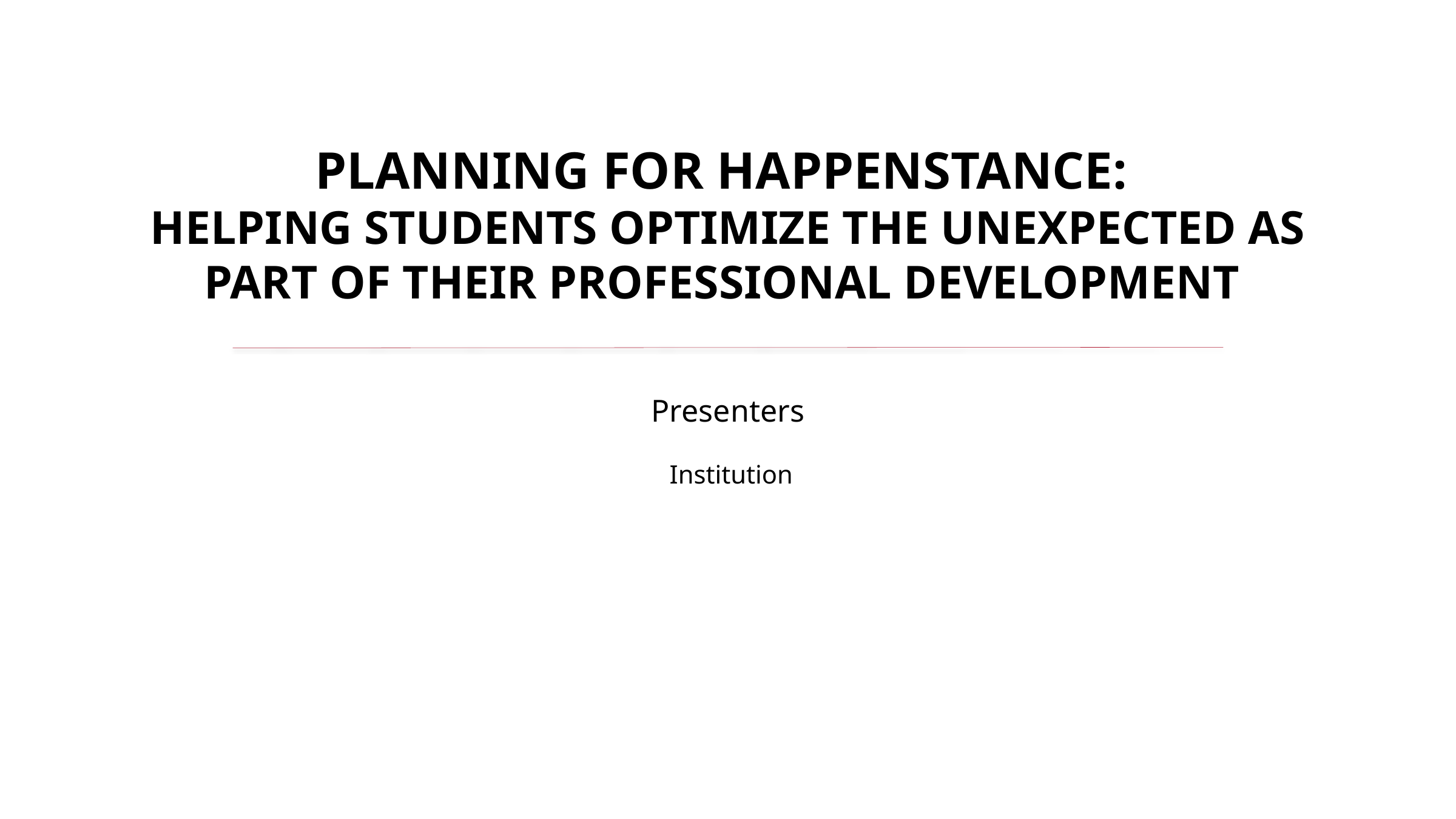

# Planning for Happenstance: Helping students optimize the unexpected as part of their professional development
Presenters
 Institution

## Slide 2
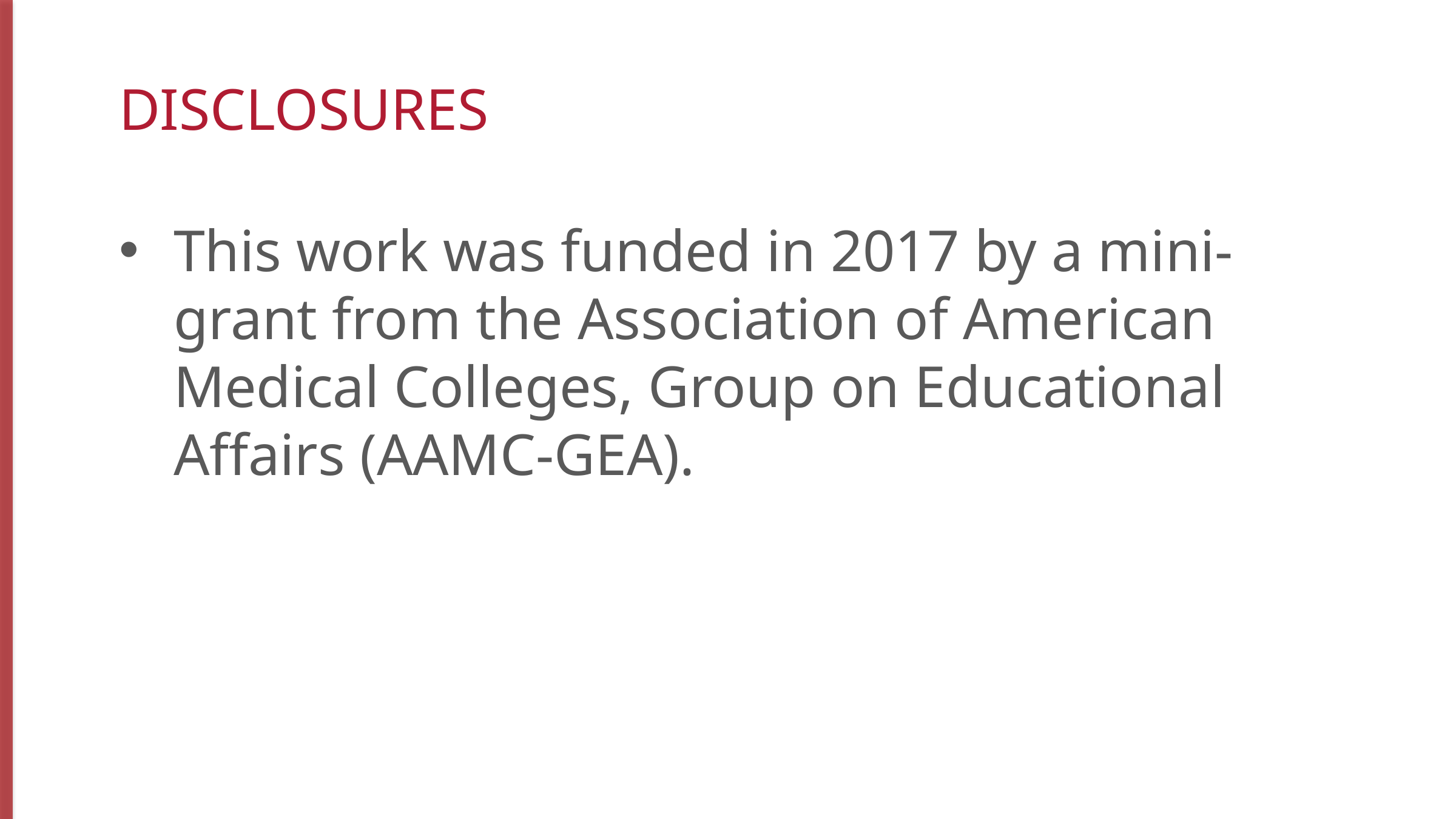

# DIsclosures
This work was funded in 2017 by a mini-grant from the Association of American Medical Colleges, Group on Educational Affairs (AAMC-GEA).

## Slide 3
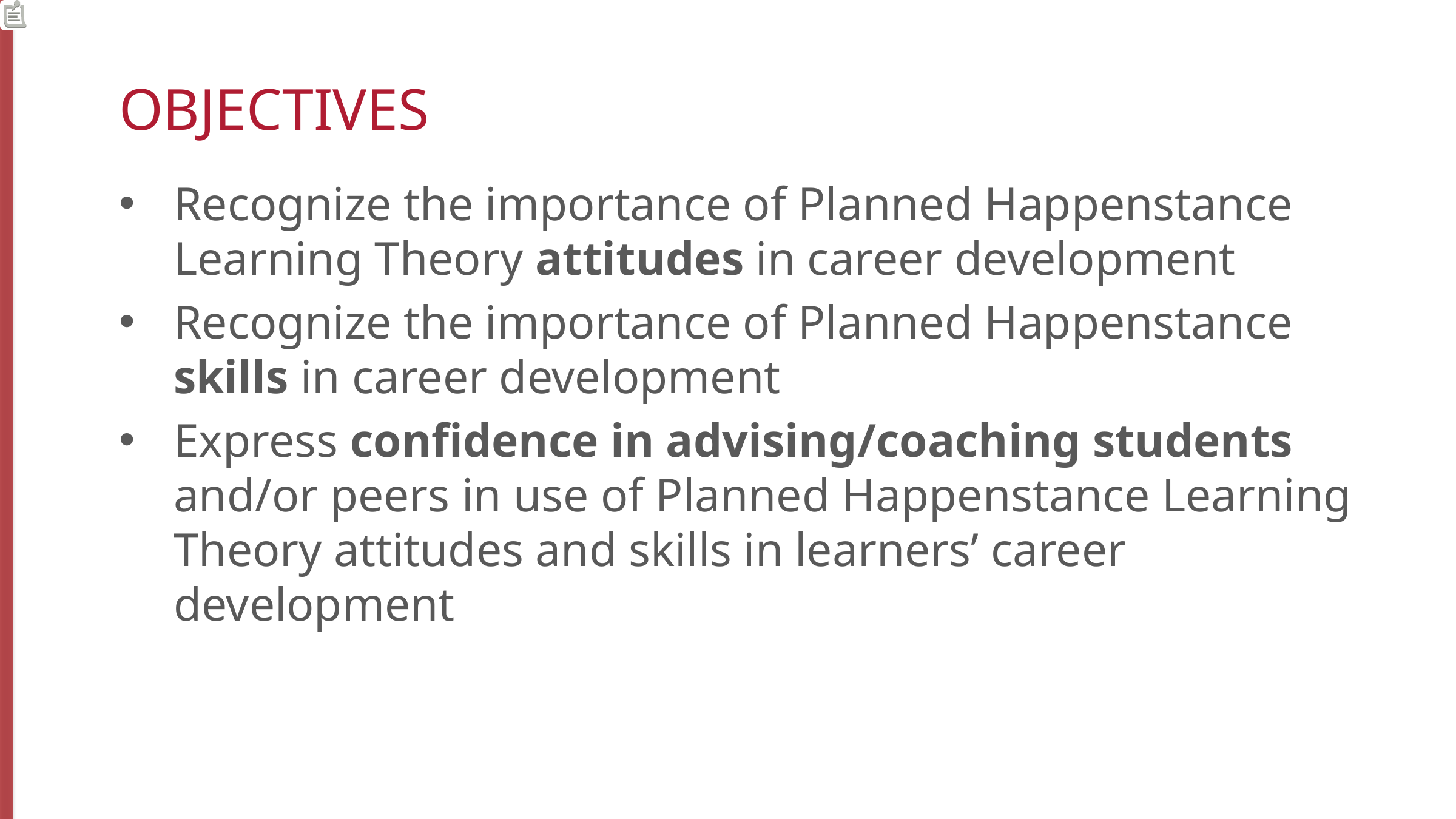

# objectives
Recognize the importance of Planned Happenstance Learning Theory attitudes in career development
Recognize the importance of Planned Happenstance skills in career development
Express confidence in advising/coaching students and/or peers in use of Planned Happenstance Learning Theory attitudes and skills in learners’ career development

## Slide 4
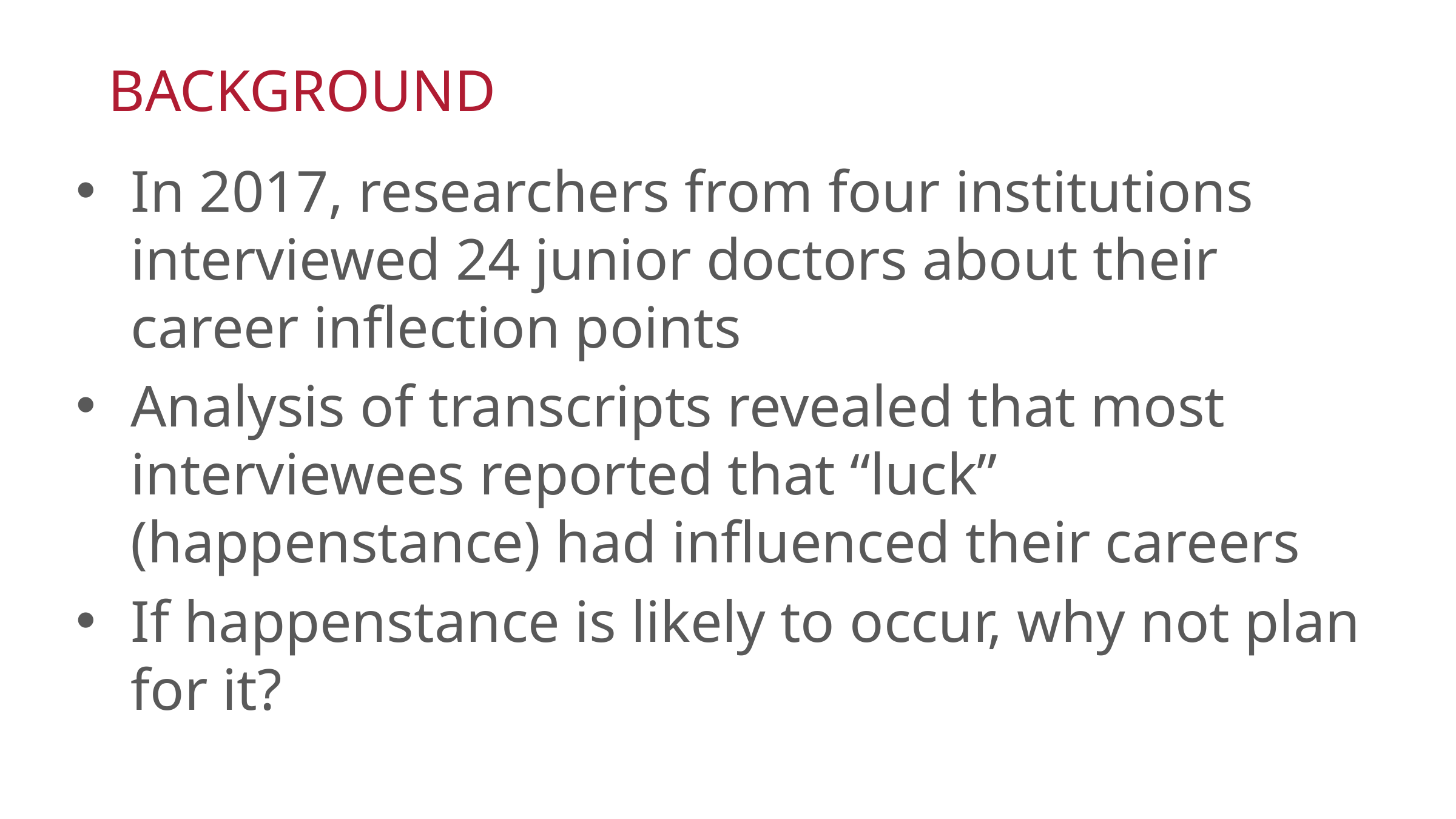

# Background
In 2017, researchers from four institutions interviewed 24 junior doctors about their career inflection points
Analysis of transcripts revealed that most interviewees reported that “luck” (happenstance) had influenced their careers
If happenstance is likely to occur, why not plan for it?

## Slide 5
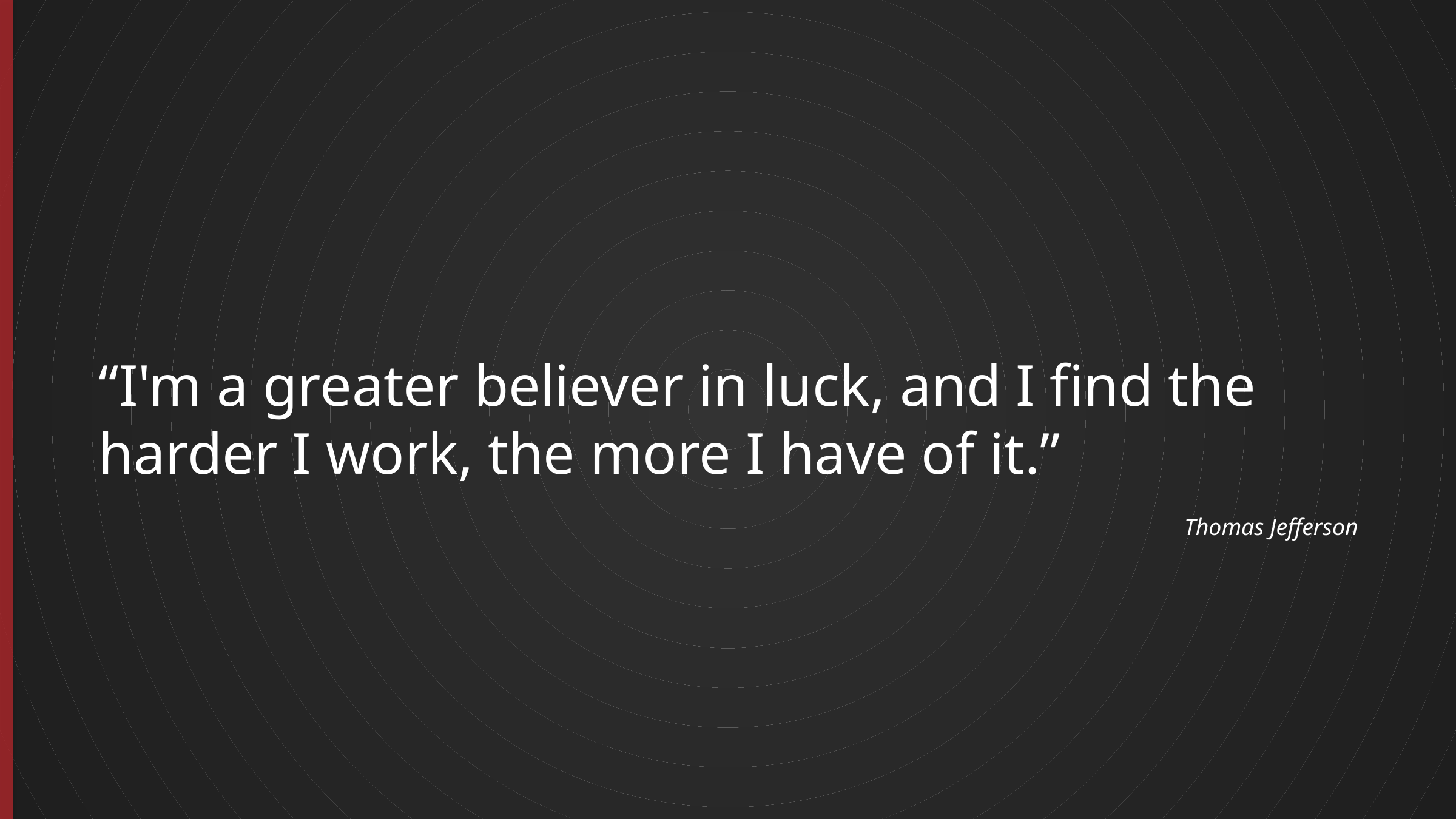

“I'm a greater believer in luck, and I find the harder I work, the more I have of it.”
Thomas Jefferson

## Slide 6
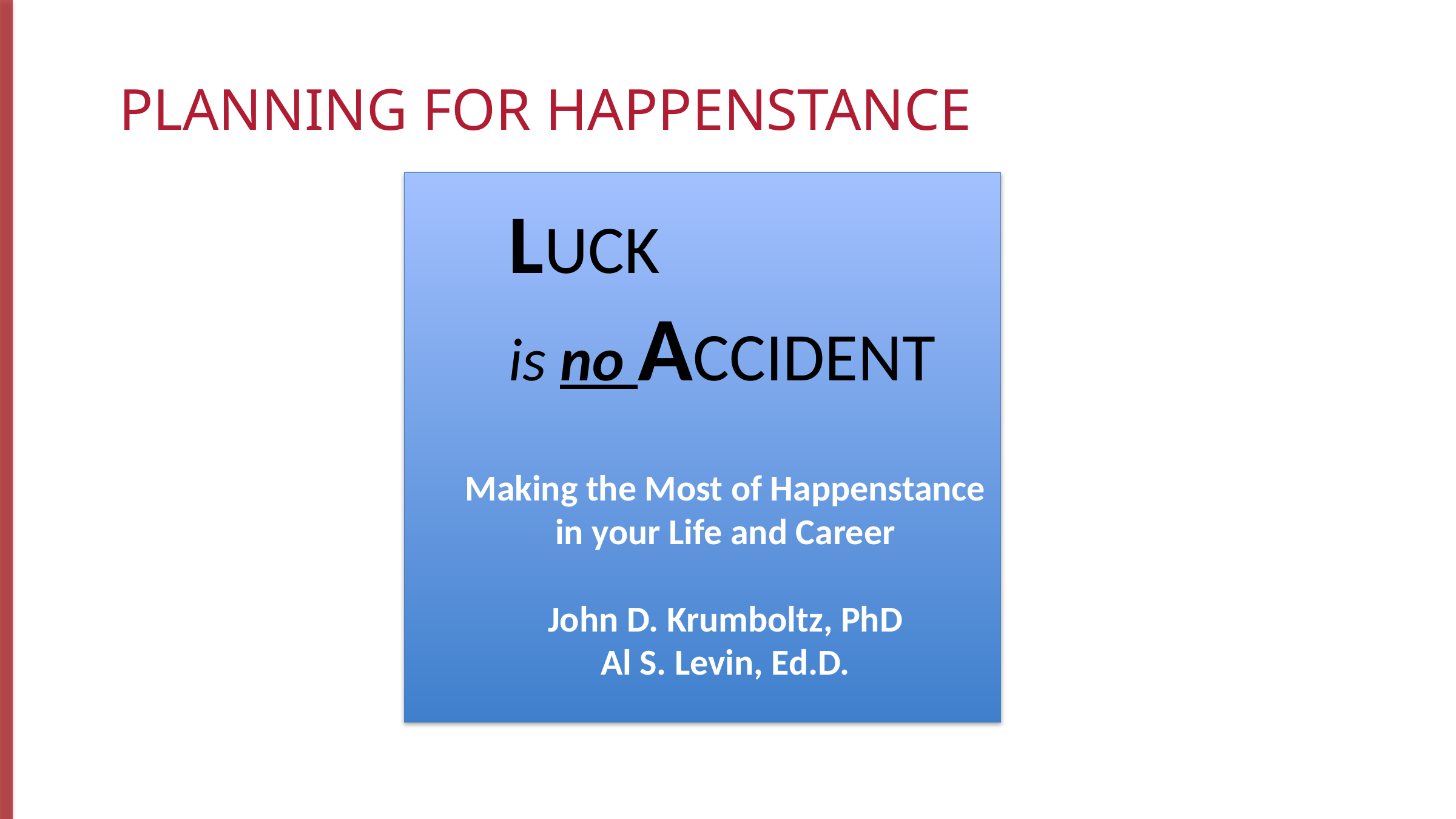

# Planning for Happenstance
LUCK
is no ACCIDENT
Making the Most of Happenstance in your Life and Career
John D. Krumboltz, PhD
Al S. Levin, Ed.D.

## Slide 7
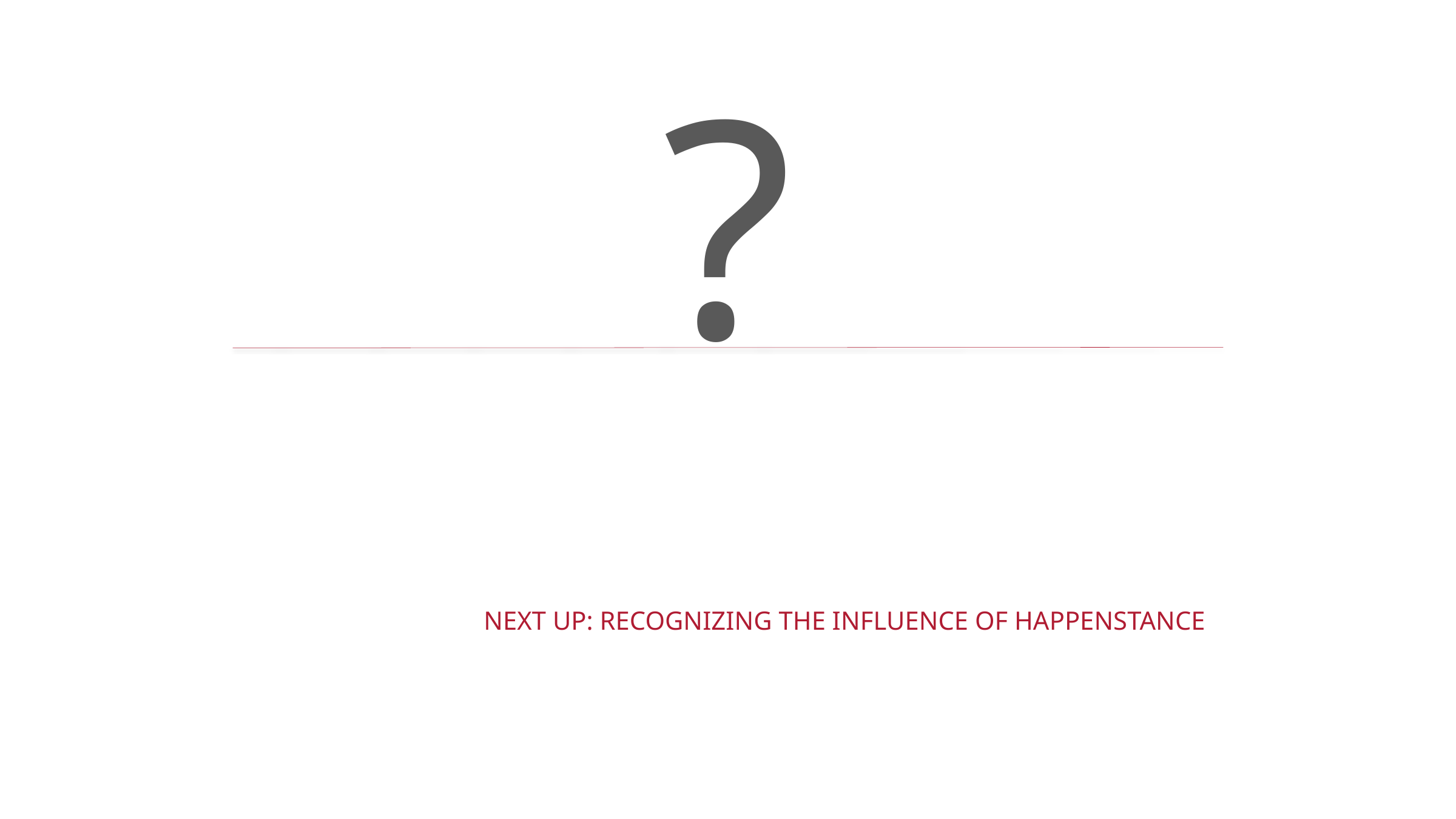

# ?
NeXT UP: Recognizing the Influence of Happenstance

## Slide 8
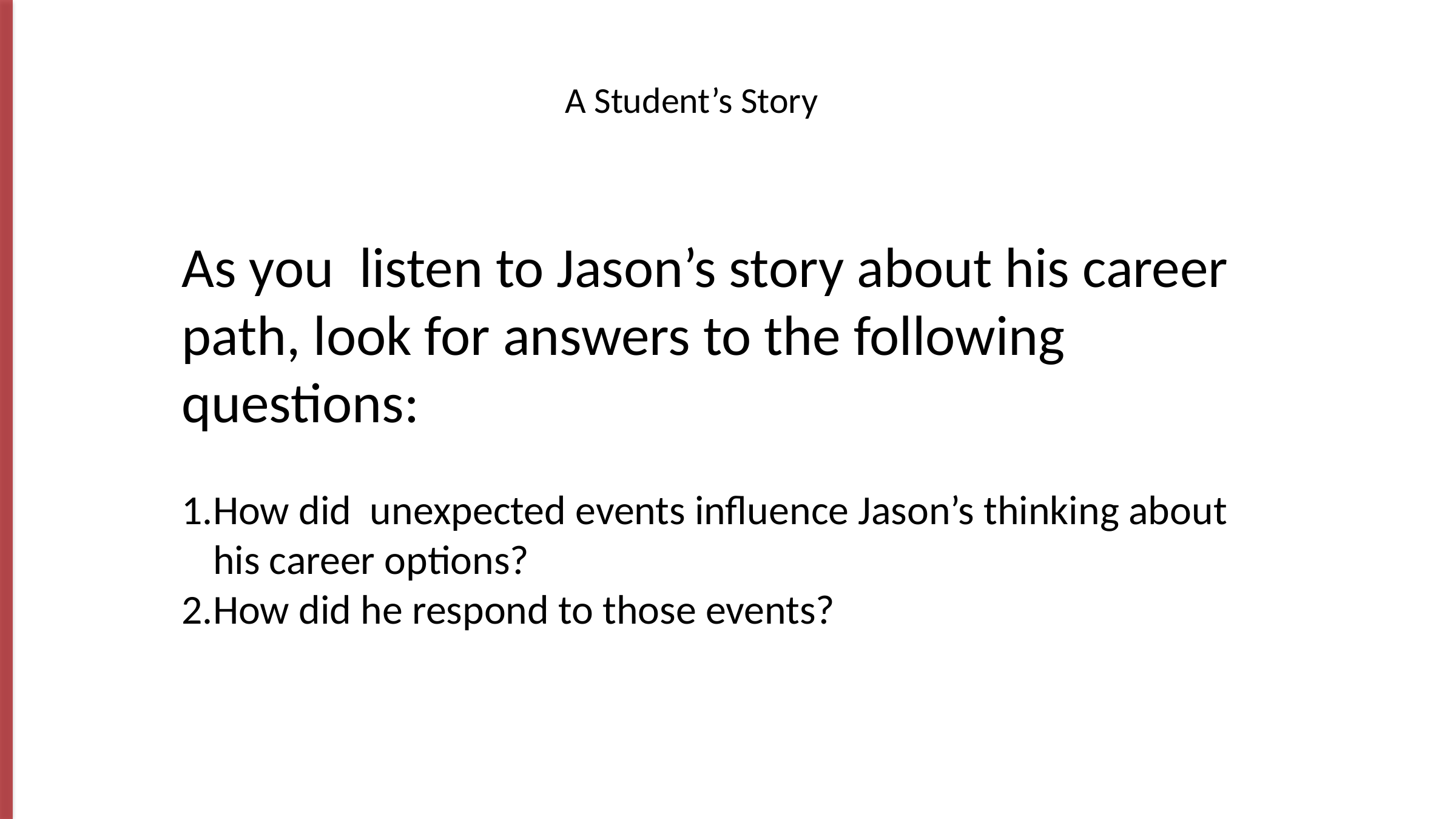

A Student’s Story
As you listen to Jason’s story about his career path, look for answers to the following questions:
How did unexpected events influence Jason’s thinking about his career options?
How did he respond to those events?

## Slide 9
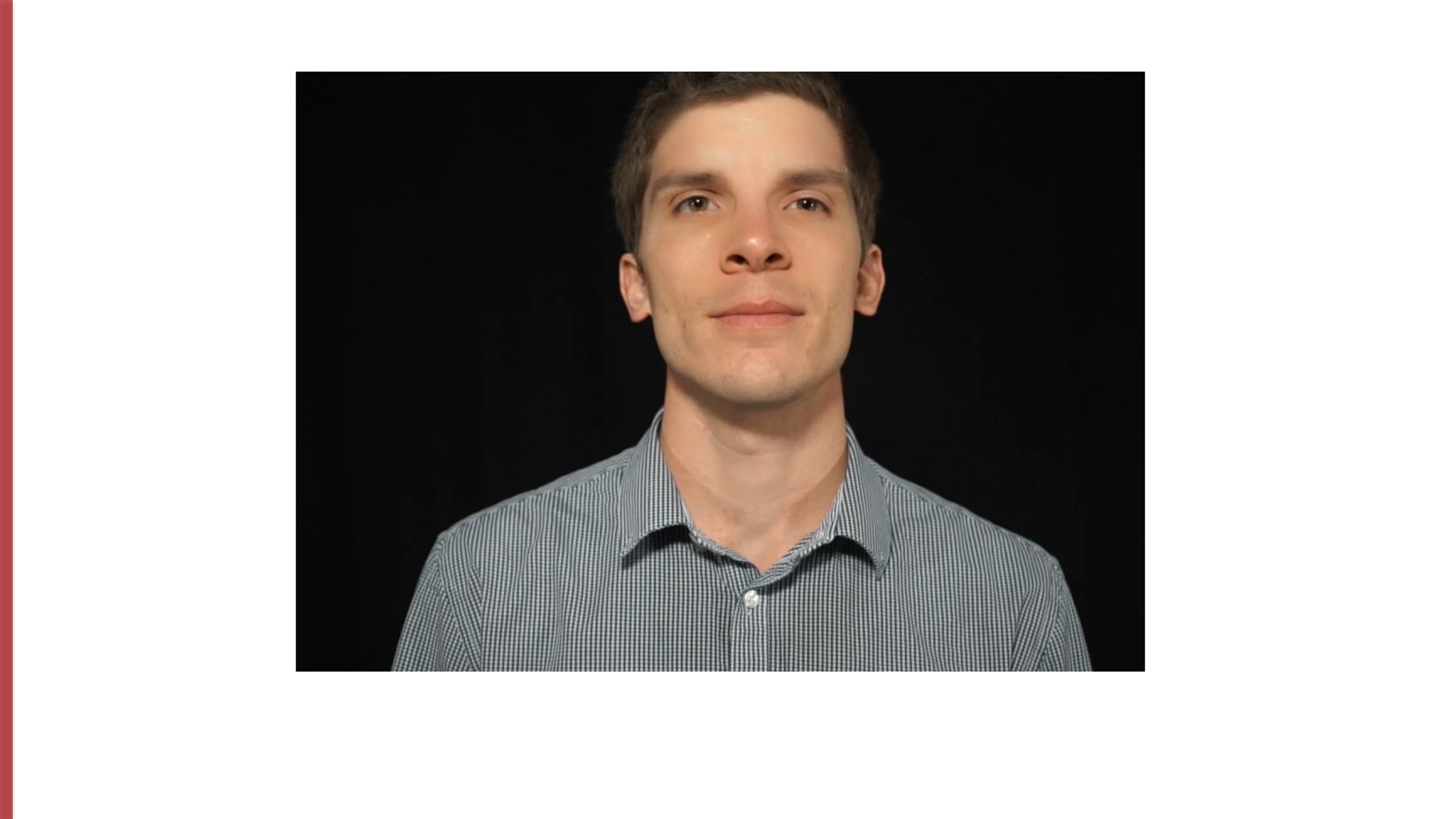

## Slide 10
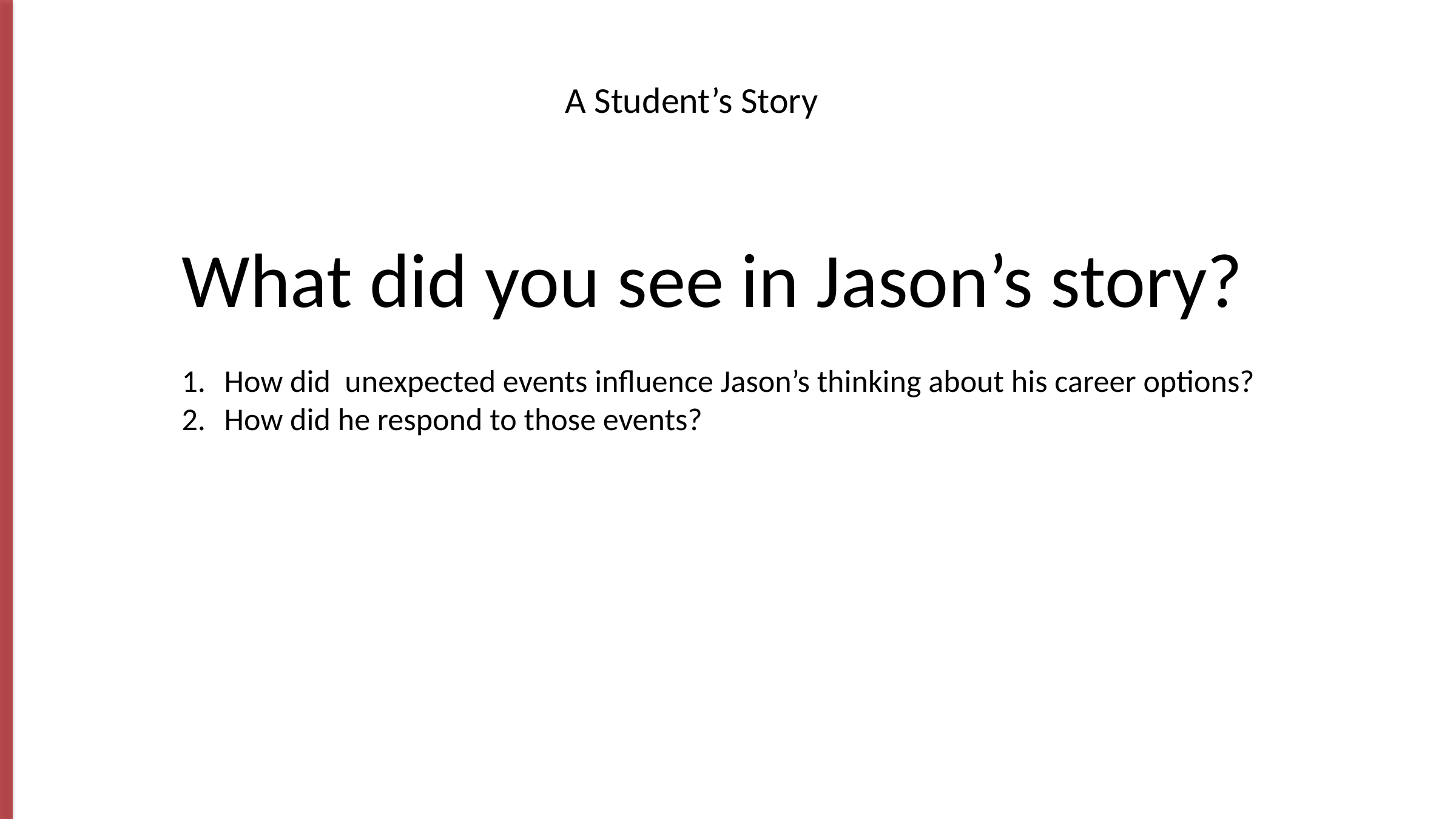

A Student’s Story
What did you see in Jason’s story?
How did unexpected events influence Jason’s thinking about his career options?
How did he respond to those events?

## Slide 11
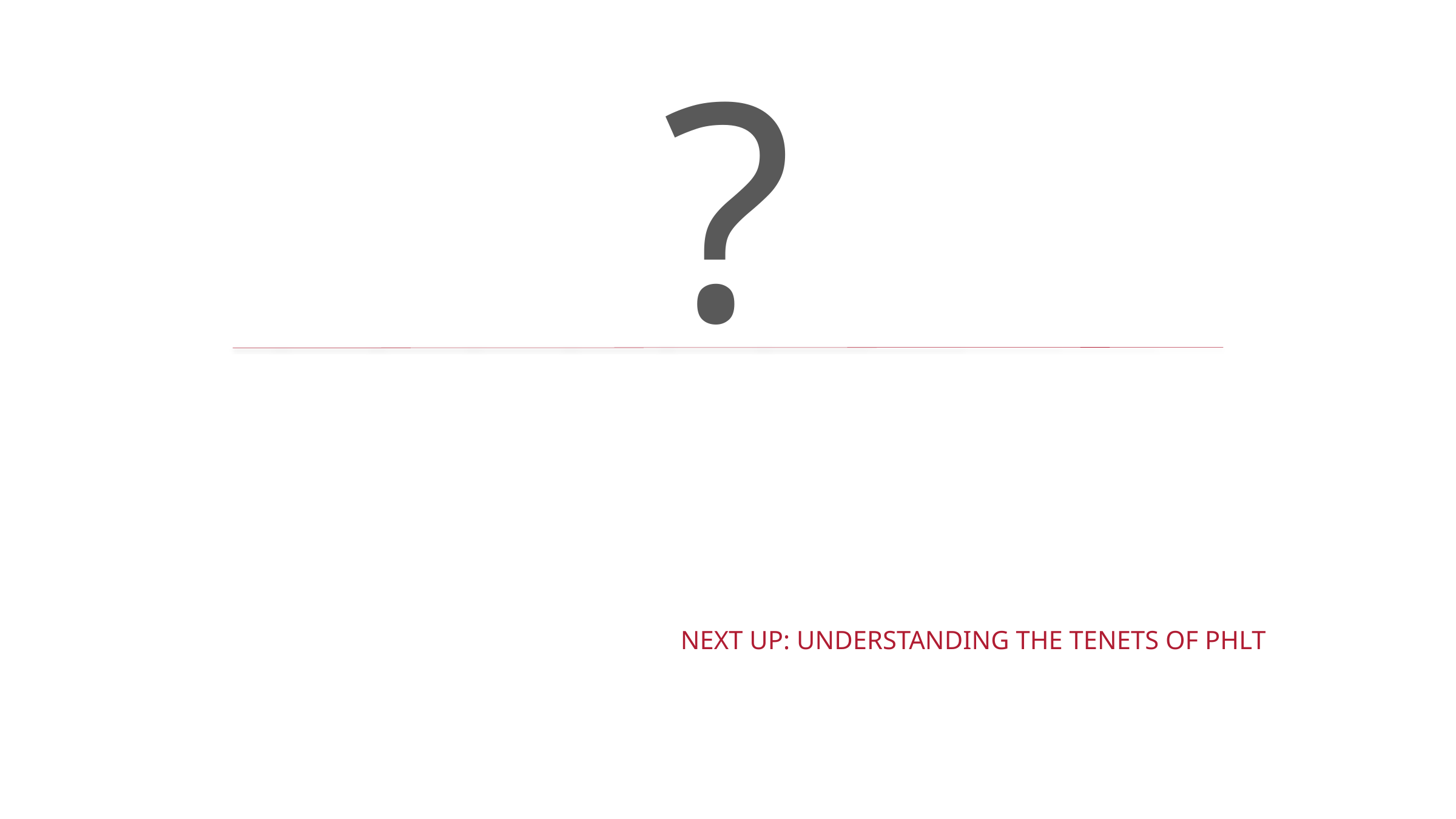

# ?
 Next up: Understanding the tenets of PHLT

## Slide 12
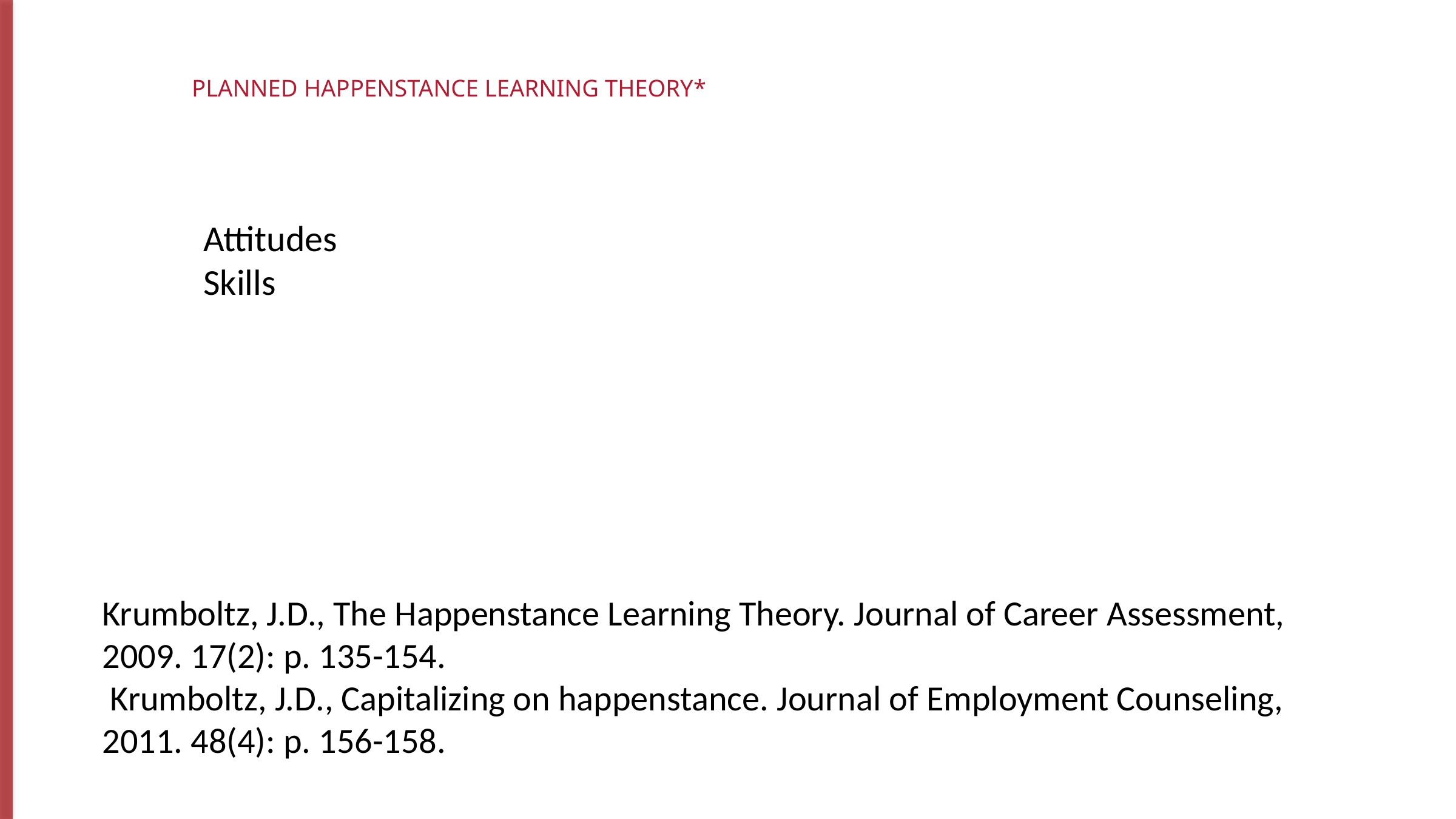

Planned Happenstance Learning Theory*
Attitudes
Skills
Krumboltz, J.D., The Happenstance Learning Theory. Journal of Career Assessment, 2009. 17(2): p. 135-154.
 Krumboltz, J.D., Capitalizing on happenstance. Journal of Employment Counseling, 2011. 48(4): p. 156-158.

## Slide 13
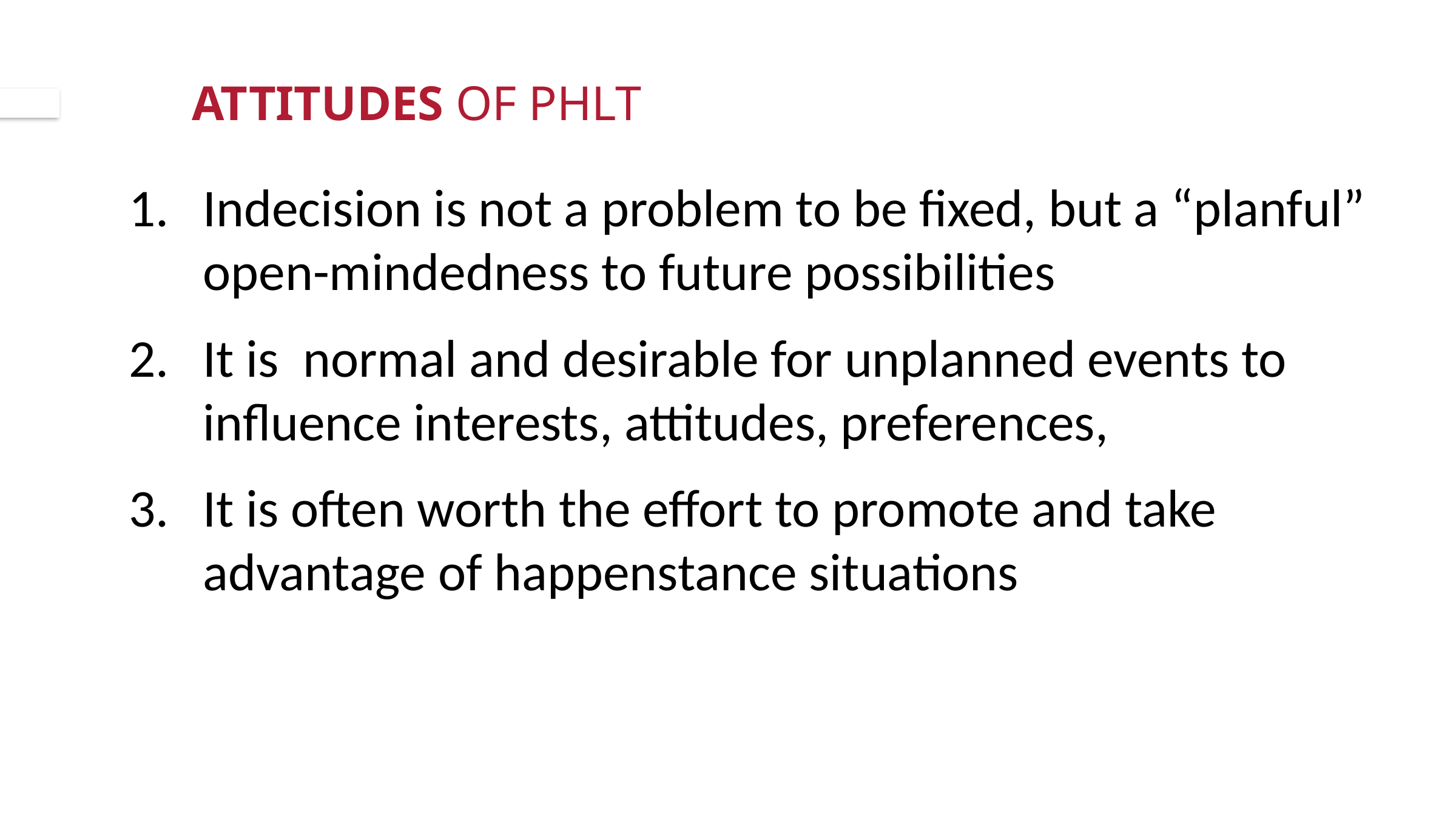

Attitudes of PHLT
Indecision is not a problem to be fixed, but a “planful” open-mindedness to future possibilities
It is normal and desirable for unplanned events to influence interests, attitudes, preferences,
It is often worth the effort to promote and take advantage of happenstance situations

## Slide 14
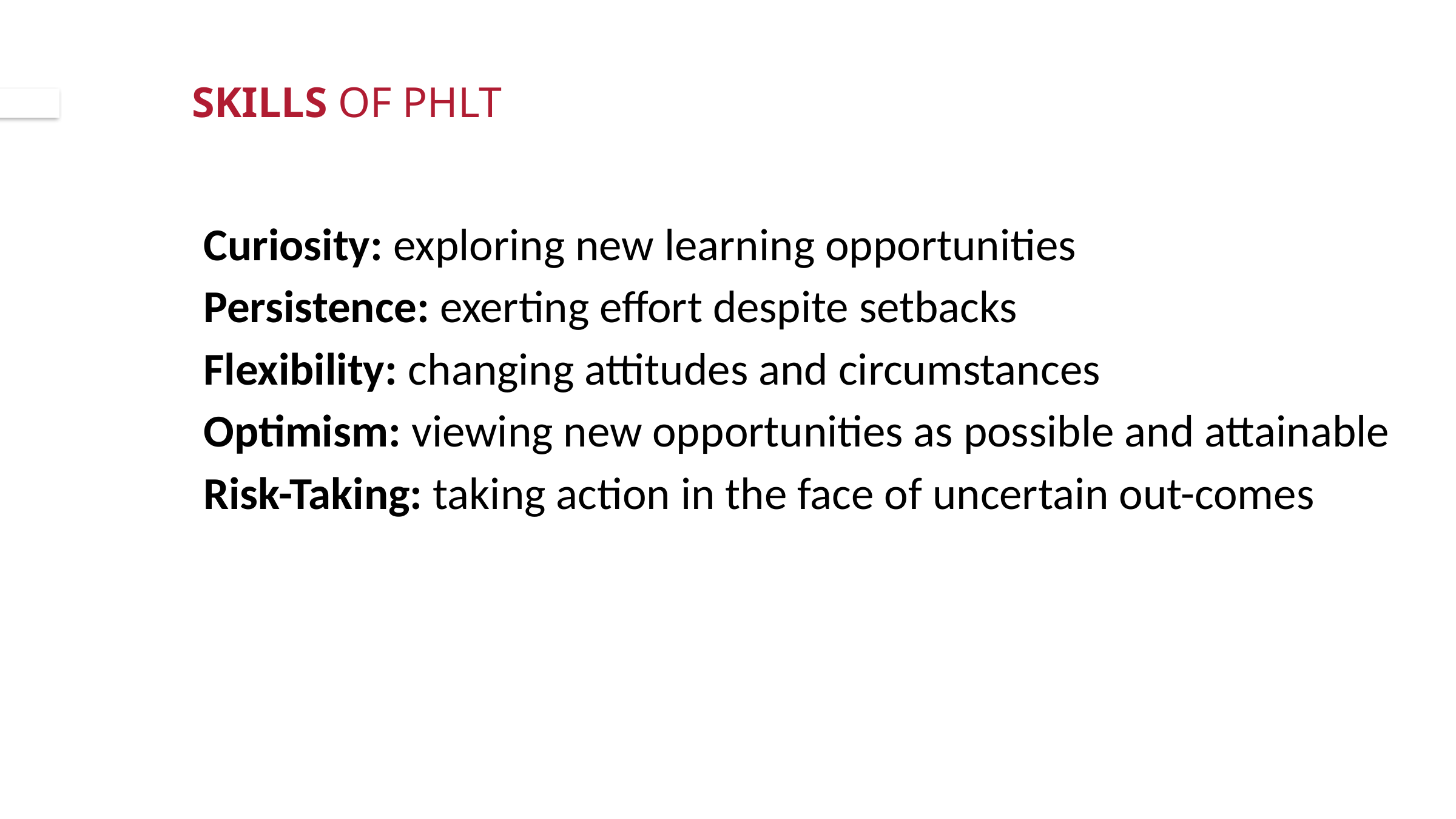

Skills of PHLT
Curiosity: exploring new learning opportunities
Persistence: exerting effort despite setbacks
Flexibility: changing attitudes and circumstances
Optimism: viewing new opportunities as possible and attainable
Risk-Taking: taking action in the face of uncertain out-comes

## Slide 15
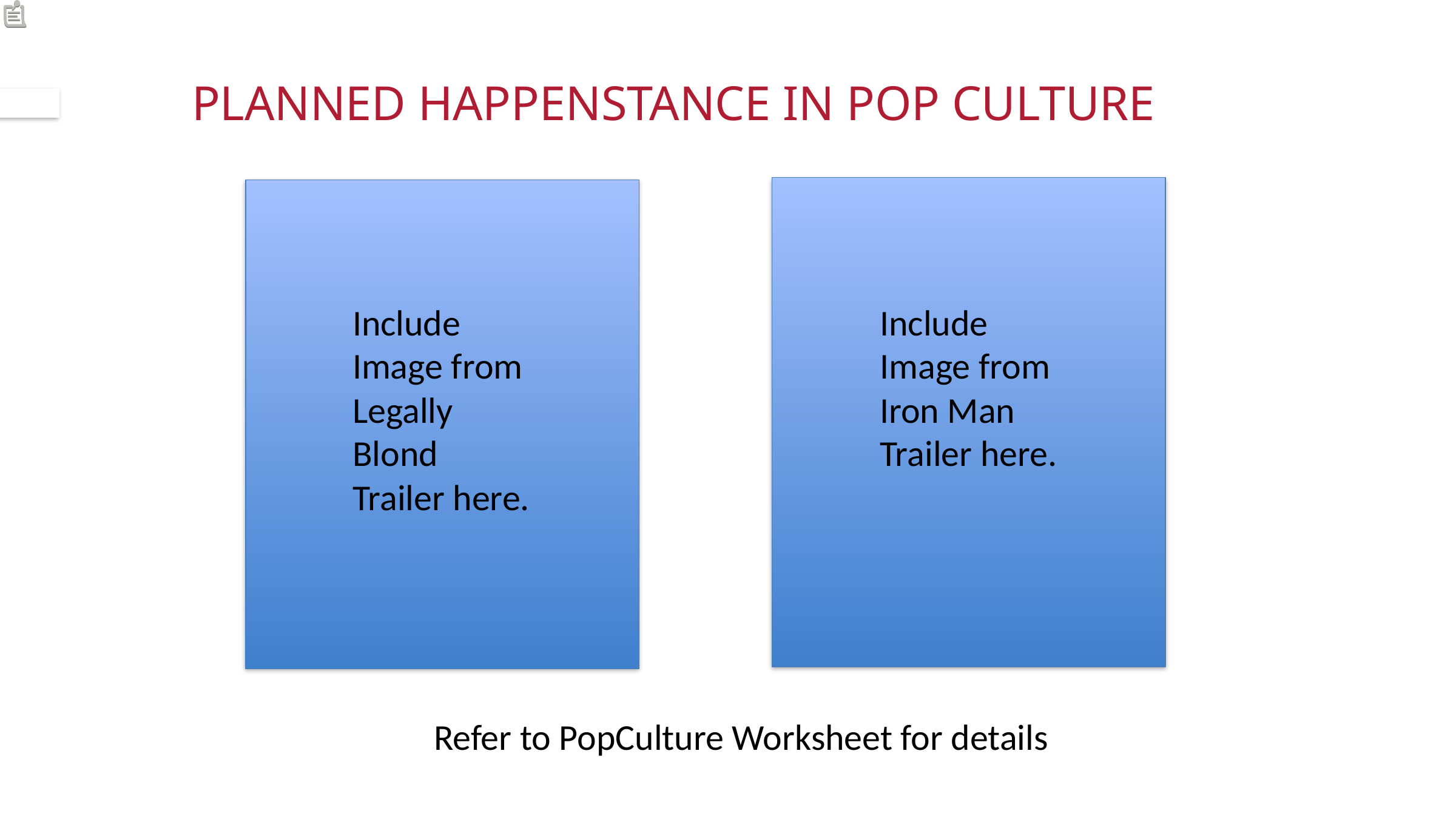

Planned happenstance in pop culture
Include Image from Legally Blond Trailer here.
Include Image from Iron Man Trailer here.
Refer to PopCulture Worksheet for details

## Slide 16
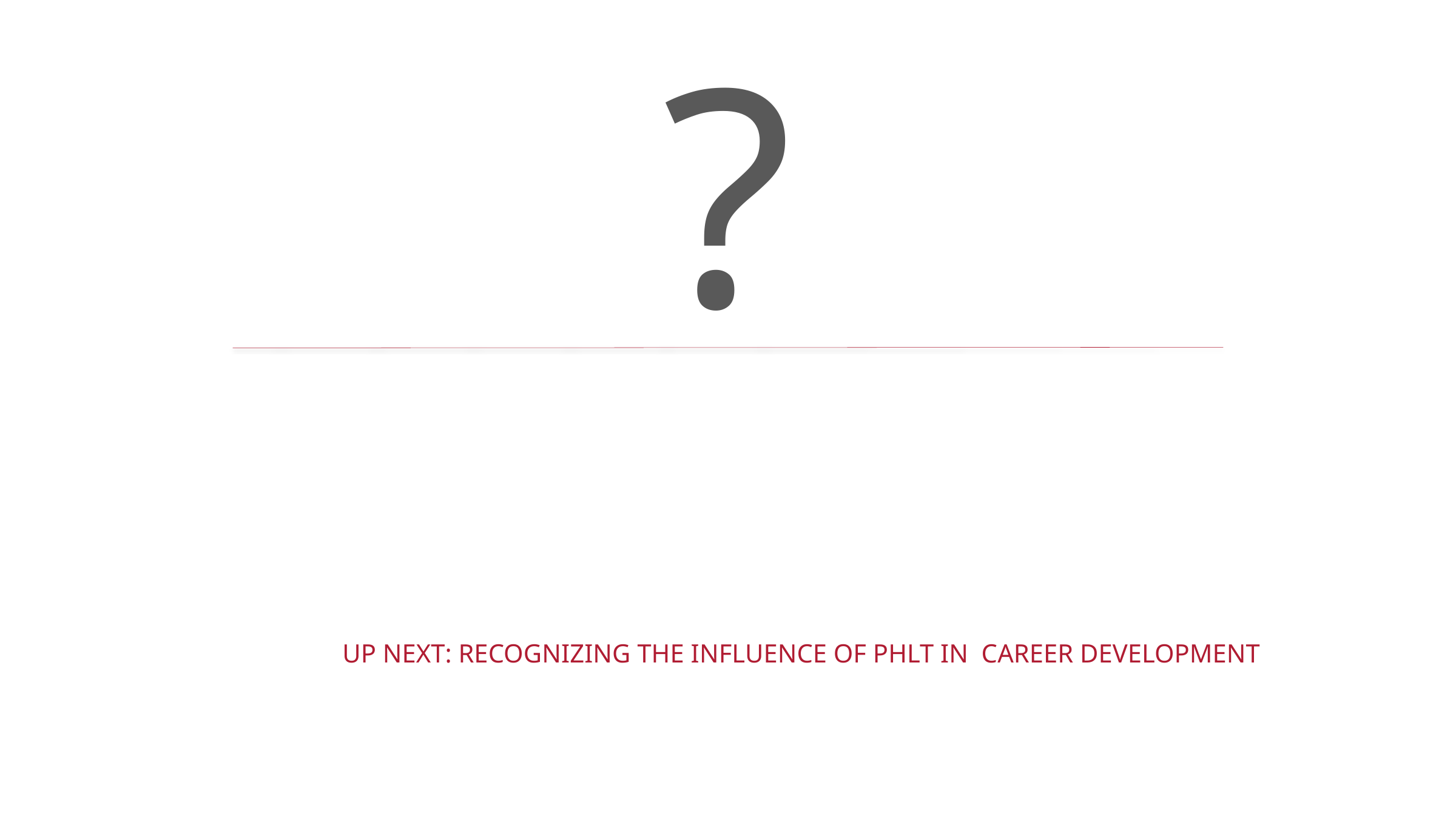

# ?
Up next: Recognizing the influence of PHLT in Career Development

## Slide 17
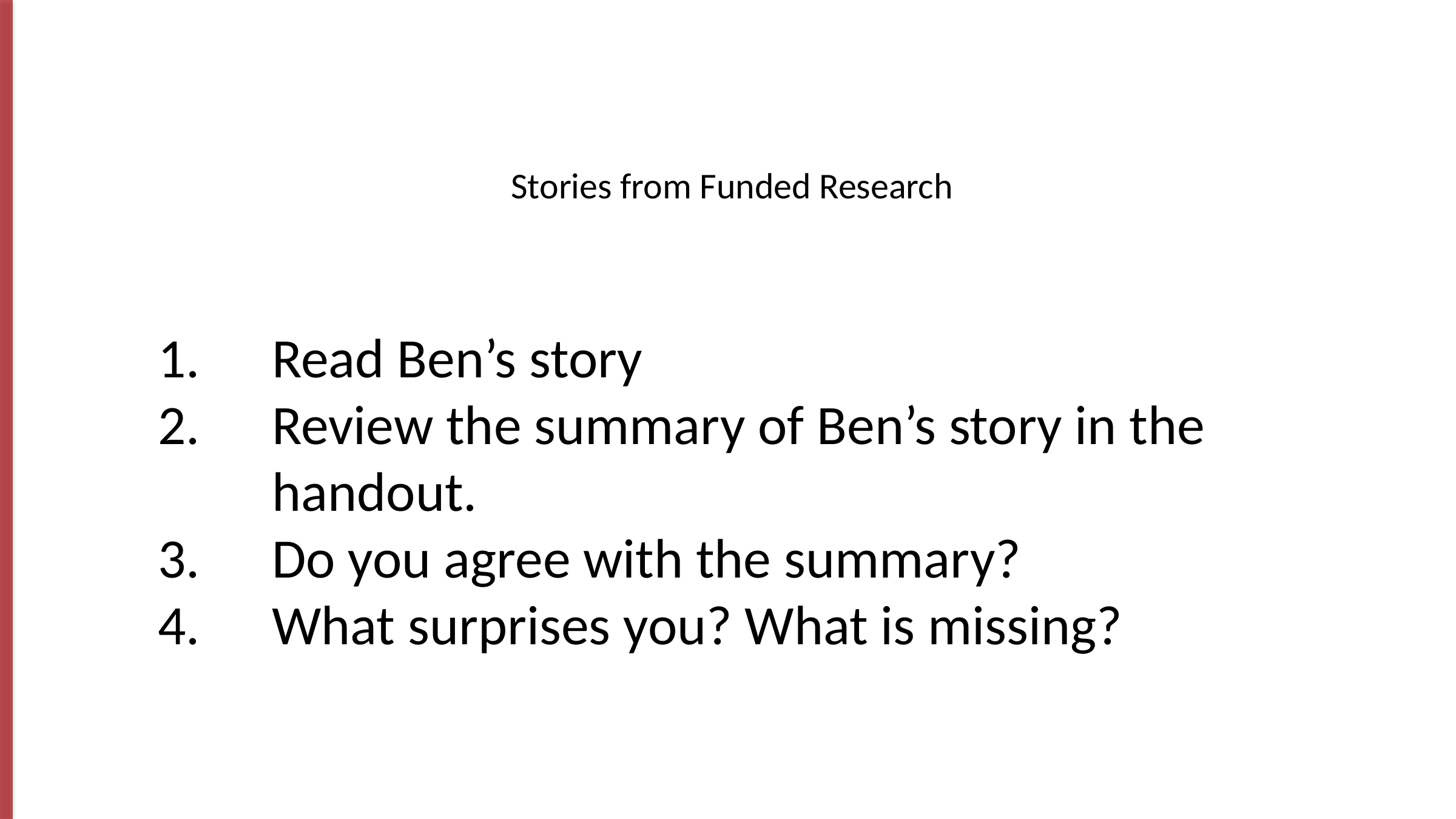

Stories from Funded Research
Read Ben’s story
Review the summary of Ben’s story in the handout.
Do you agree with the summary?
What surprises you? What is missing?

## Slide 18
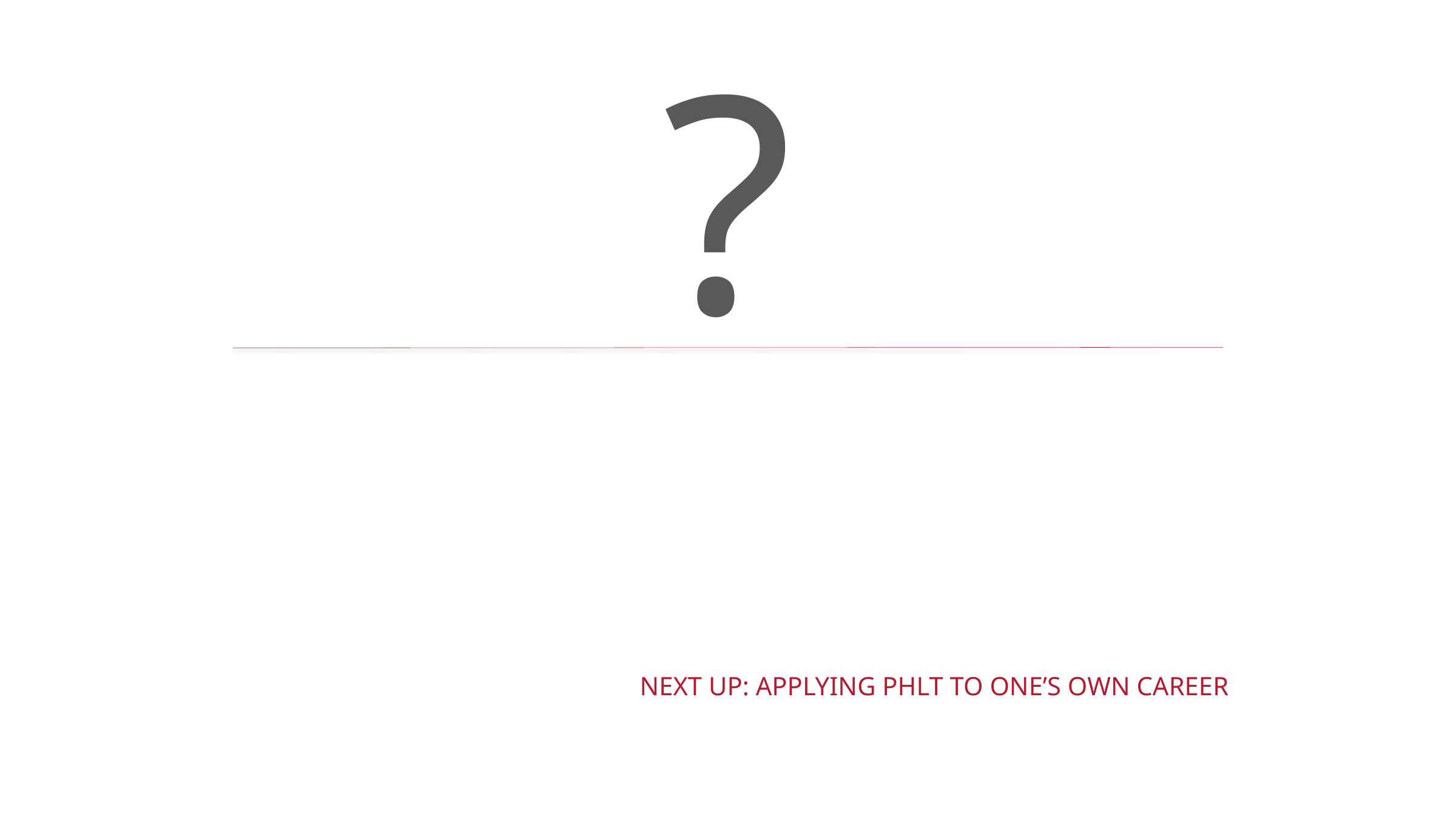

# ?
Next up: Applying PHLT to one’s own career

## Slide 19
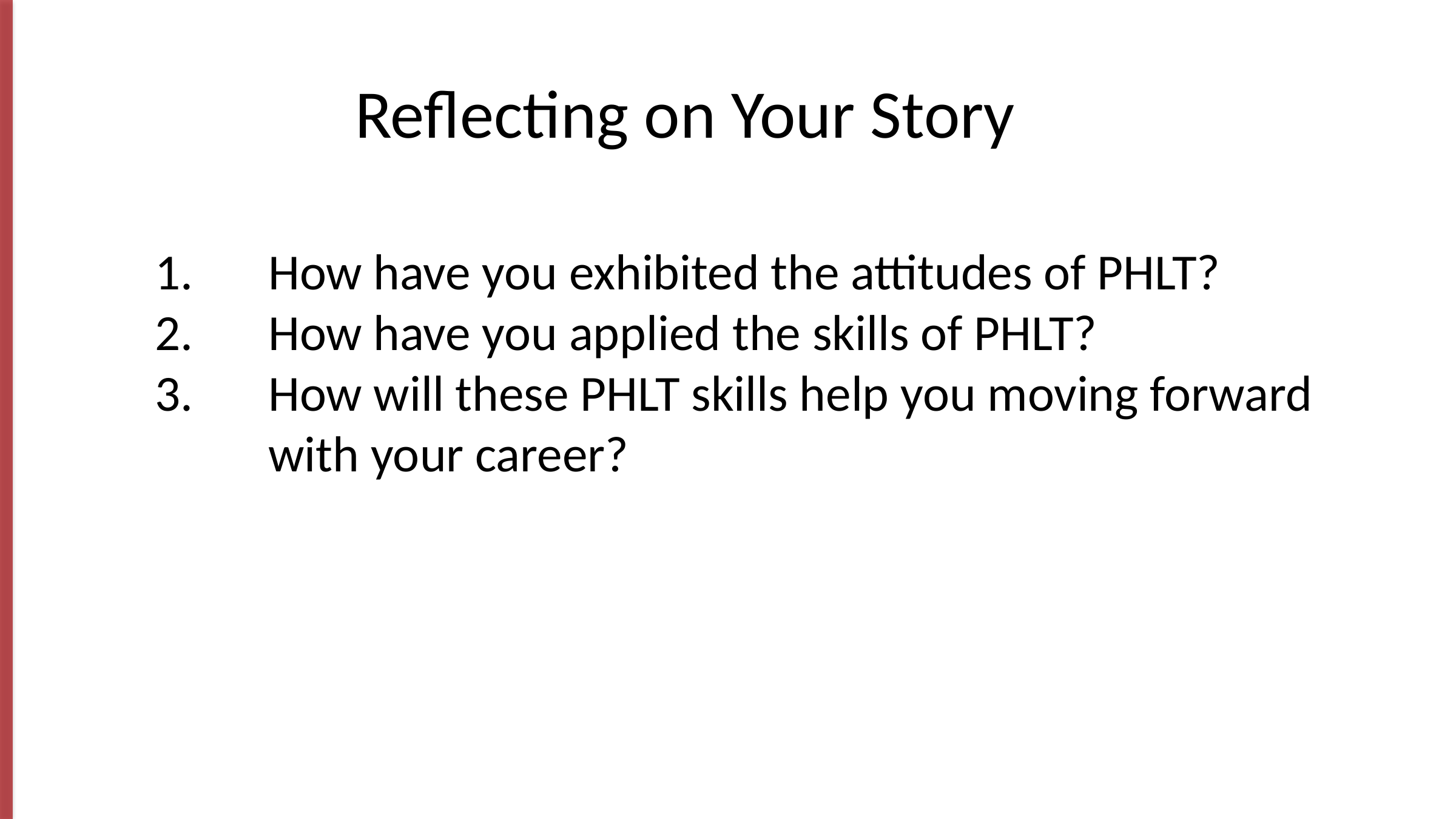

Reflecting on Your Story
How have you exhibited the attitudes of PHLT?
How have you applied the skills of PHLT?
How will these PHLT skills help you moving forward with your career?

## Slide 20
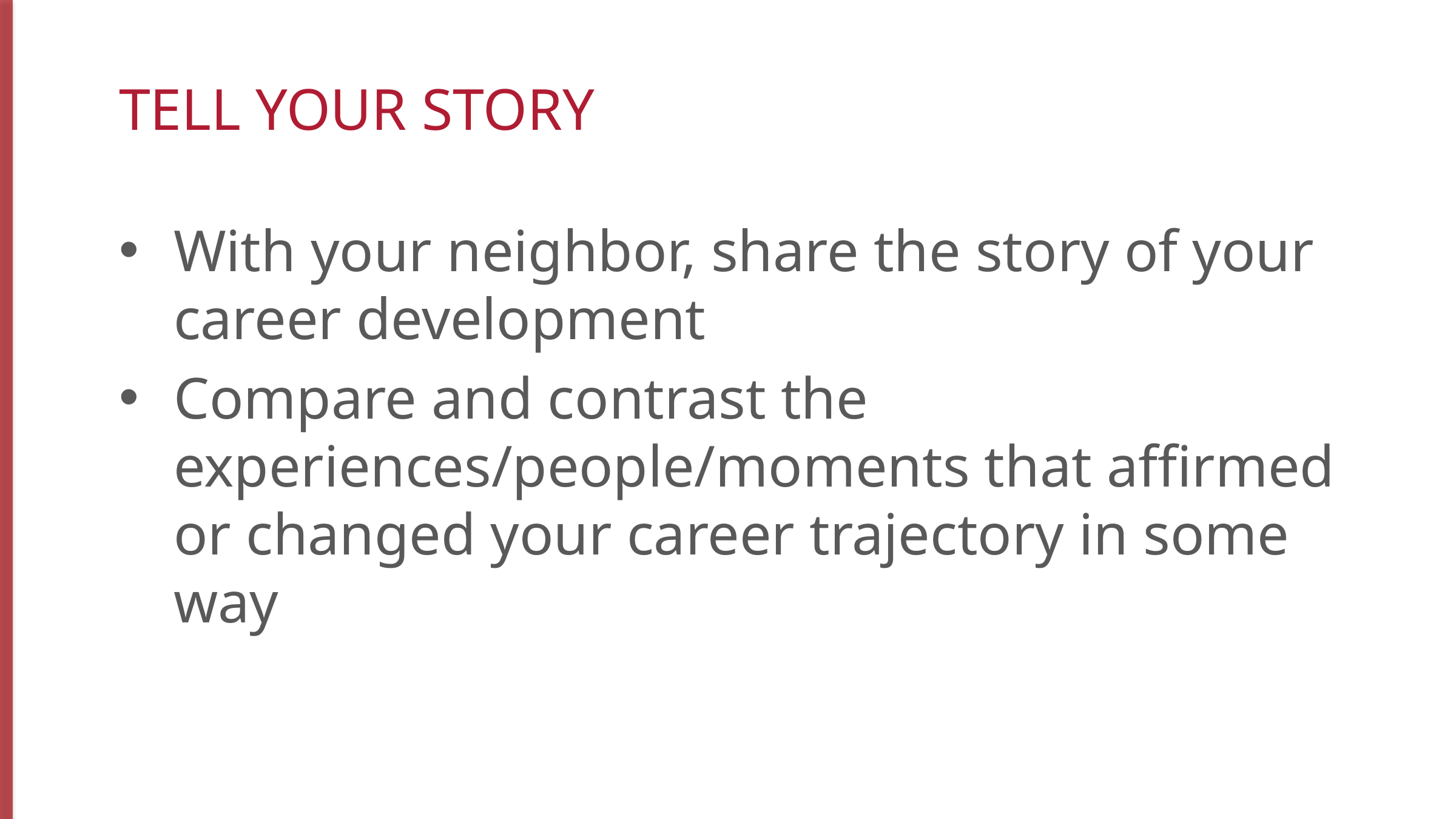

# Tell Your story
With your neighbor, share the story of your career development
Compare and contrast the experiences/people/moments that affirmed or changed your career trajectory in some way

## Slide 21
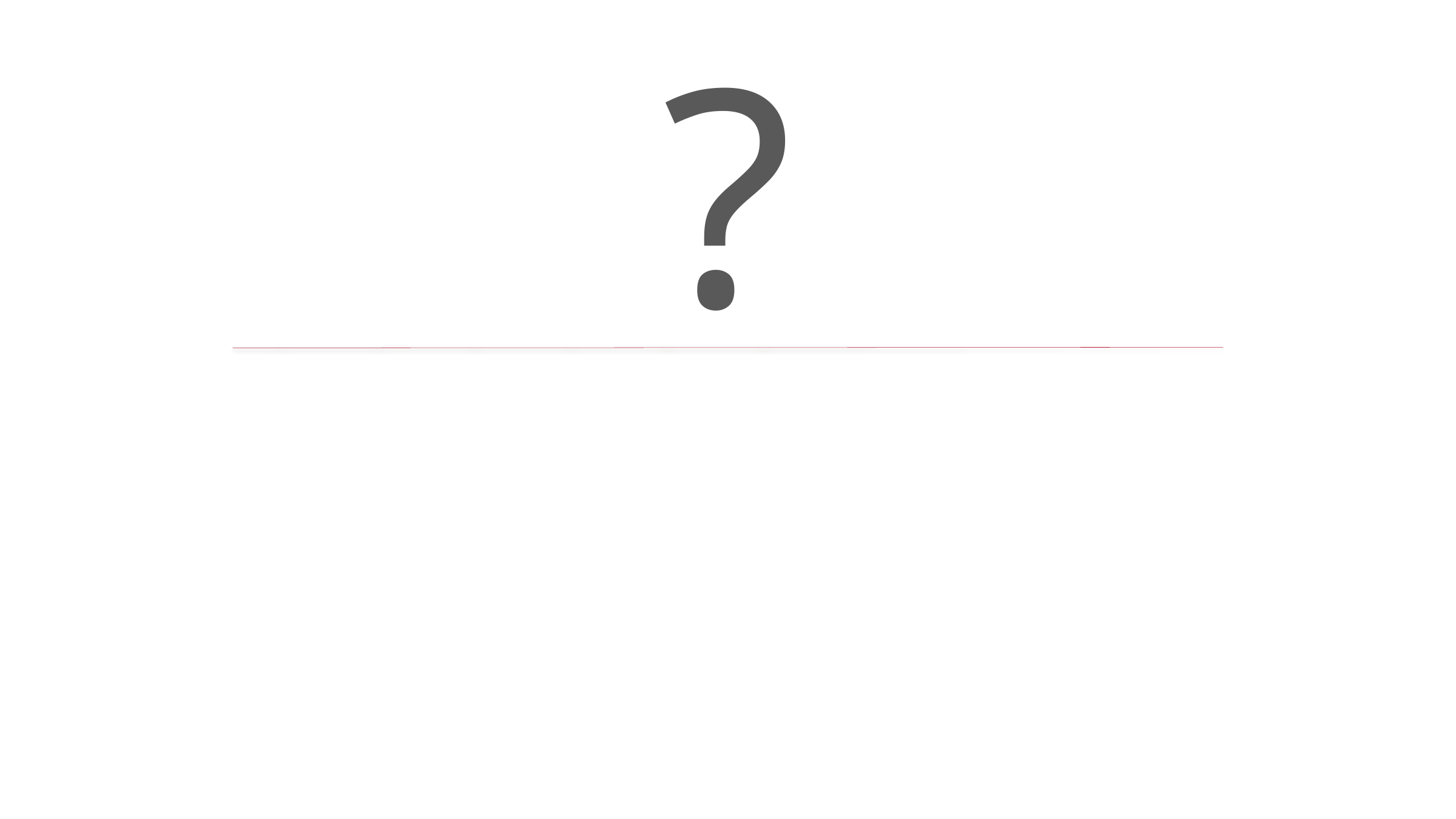

# ?

## Slide 22
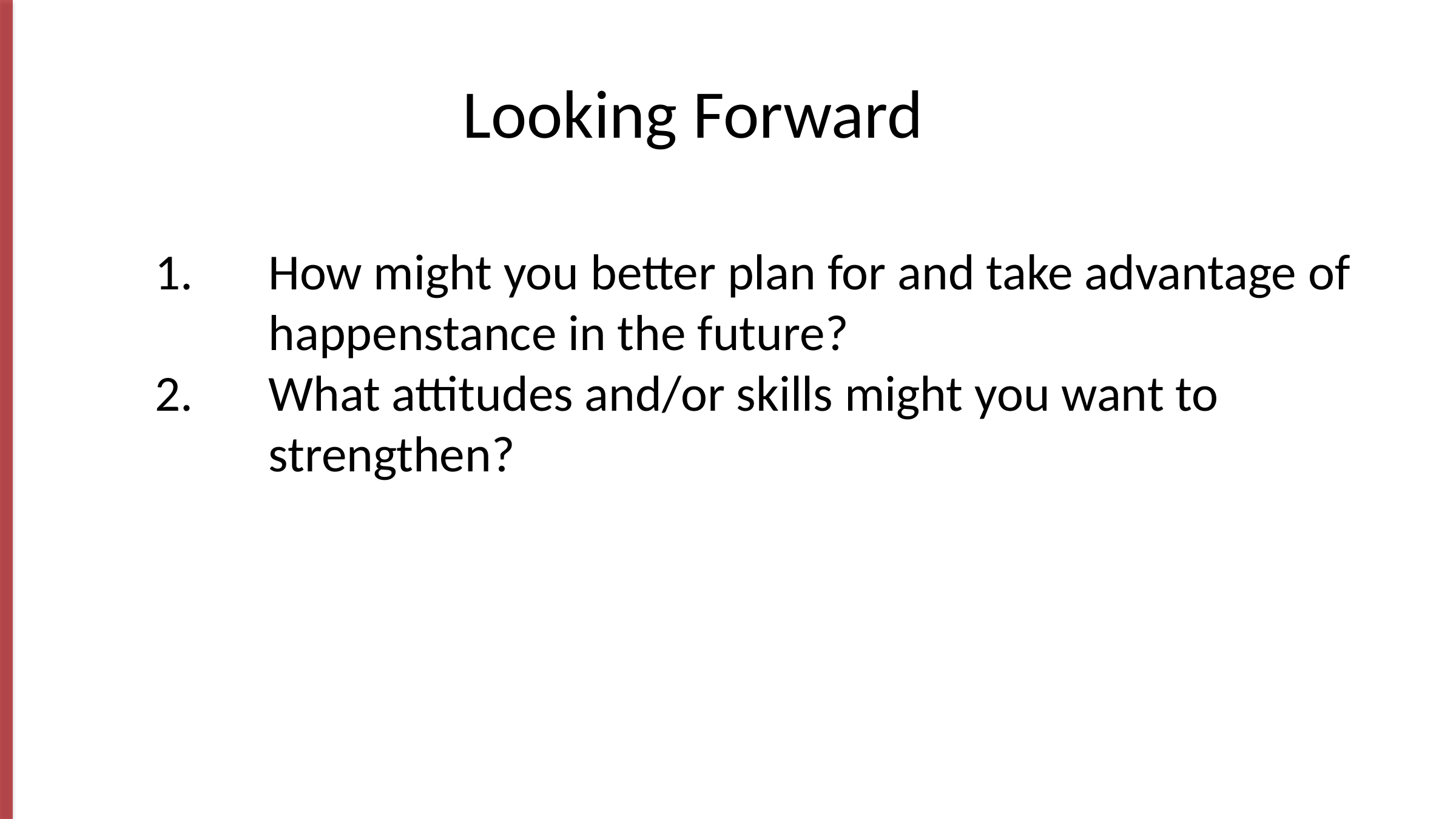

Looking Forward
How might you better plan for and take advantage of happenstance in the future?
What attitudes and/or skills might you want to strengthen?

## Slide 23
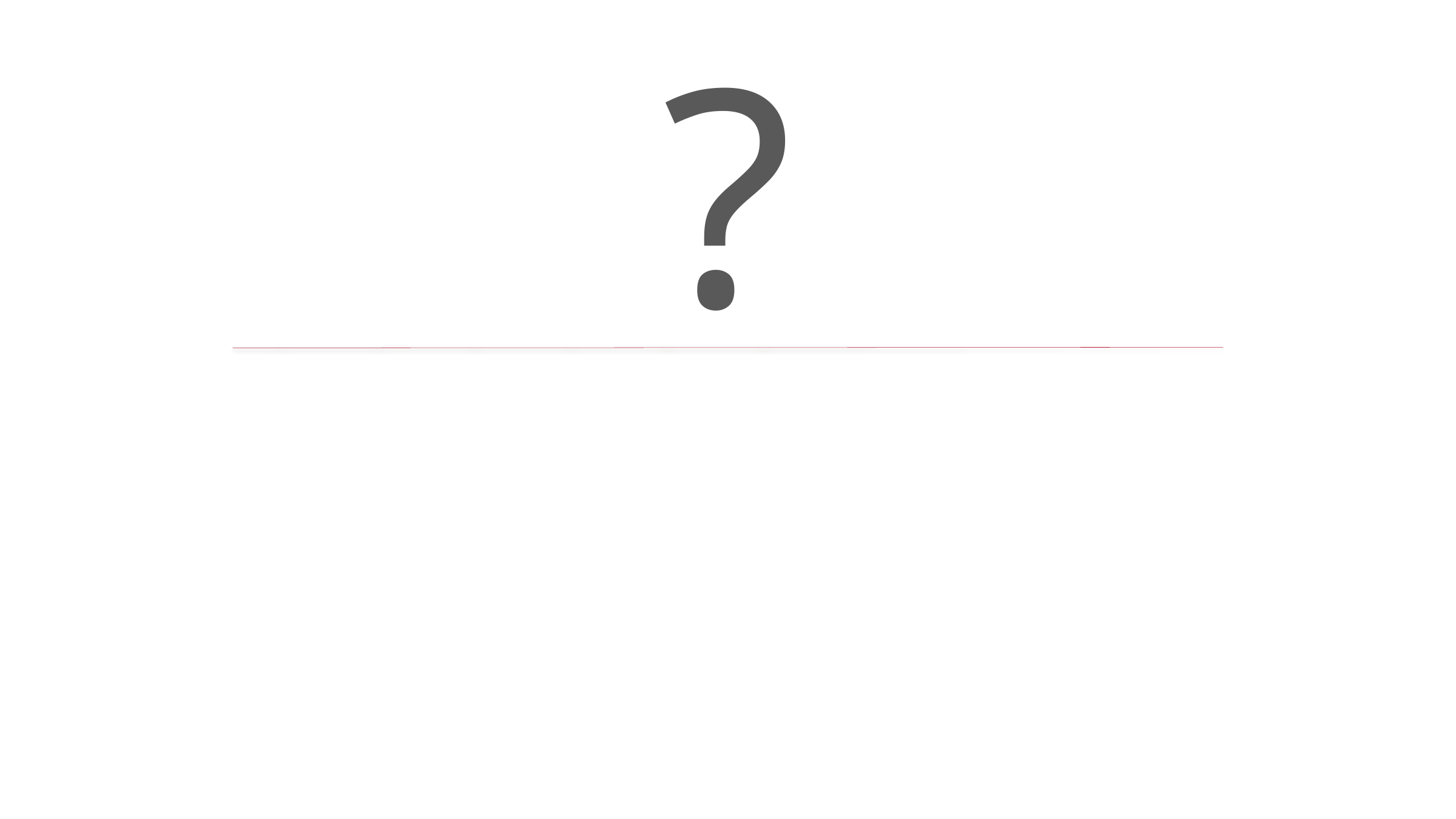

# ?

## Slide 24
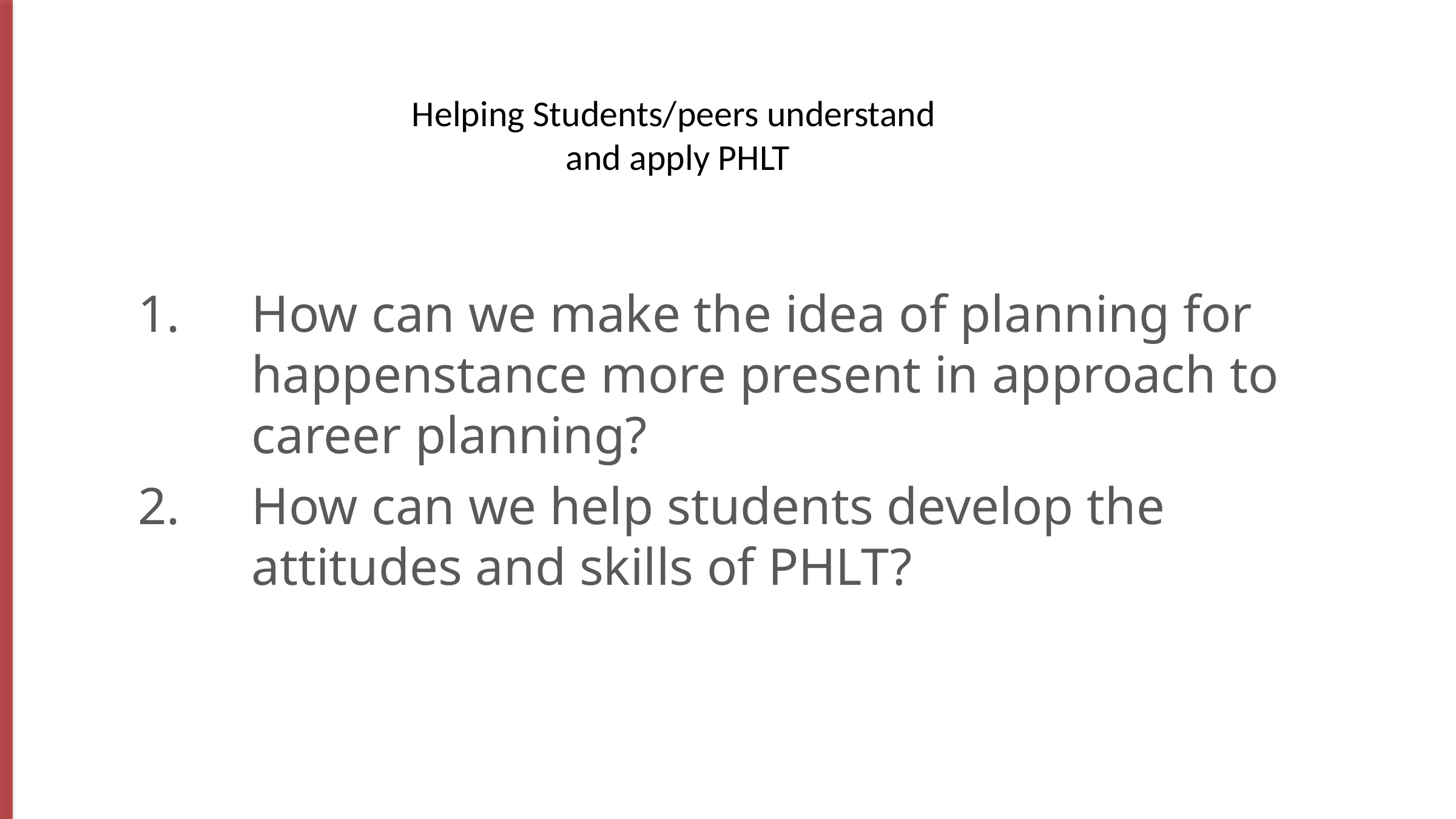

Helping Students/peers understand
and apply PHLT
How can we make the idea of planning for happenstance more present in approach to career planning?
How can we help students develop the attitudes and skills of PHLT?

## Slide 25
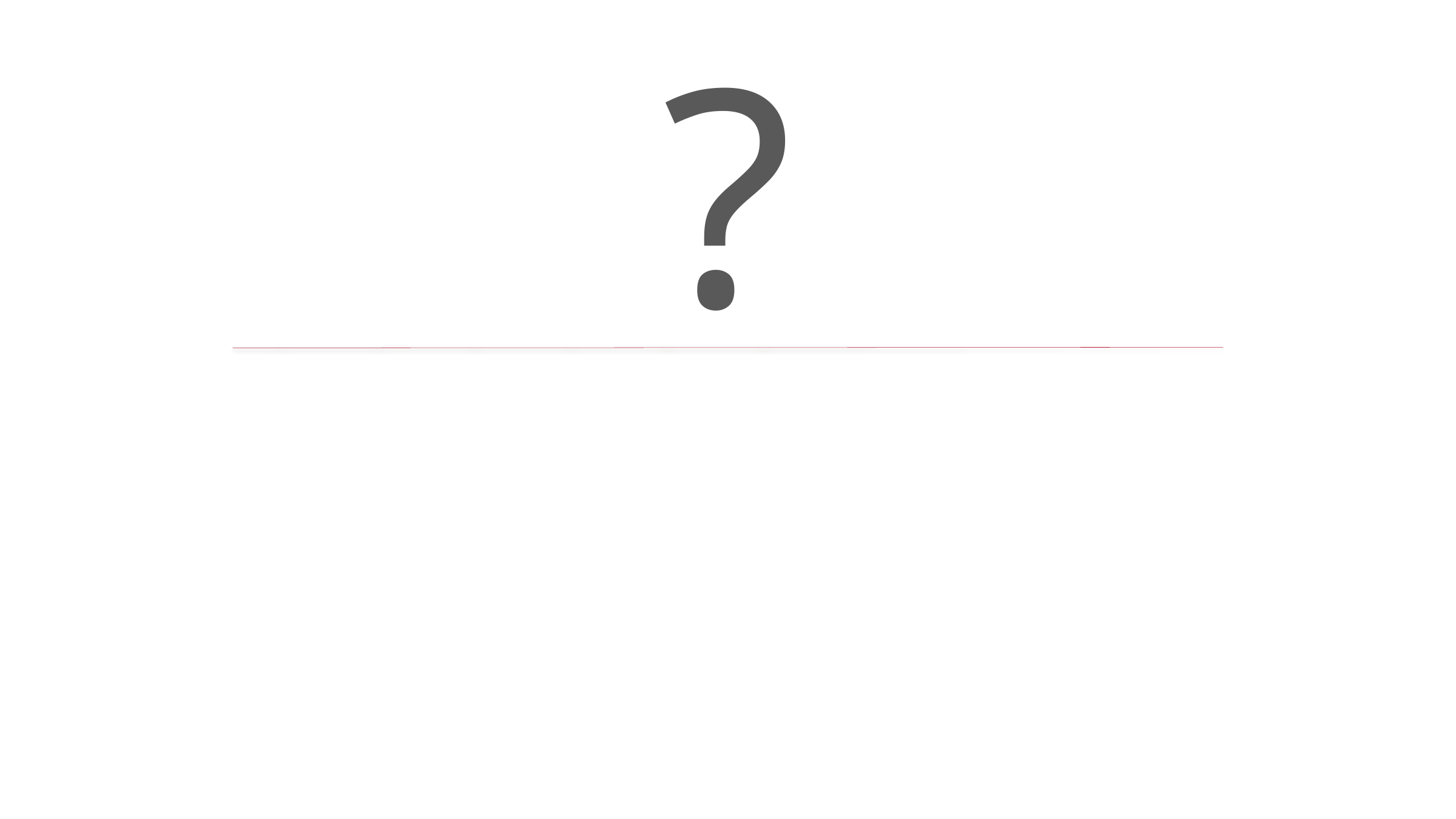

# ?
