# Supplementary material for: Planning for Happenstance: Helping Students Optimize Unexpected Career Developments
Source: MedEdPORTAL. 2021 Feb 8;17:11087. doi: 10.15766/mep_2374-8265.11087 (PMC7880249; doi:10.15766/mep_2374-8265.11087)
Supplement: Supplementary file 1 — Eight Stories.docInstructor Guide.docPowerPoint Slides.pptxJason's Story PHLT Video.mp4PHLT Worksheet.docxPHLT Workshop Evaluation.docx [file mep_2374-8265.11087-s001.zip › E. PHLT Worksheet.docx]

**Planned Happenstance Learning Theory Worksheet**

| **Attitudes** | **Legally Blond Story** | **Iron Man Story** |
| --- | --- | --- |
| Indecision is not a problem to be fixed, but **a “planful” open-mindedness** to future possibilities. | Elle wasn’t sure if she wanted to stay at Harvard after her professor made a pass at her, but she was open-minded to the advice of others and ended up staying and winning a case. | Tony Stark was willing to change his mind and announce his identity as Iron Man in order to create more future possibilities. |
| It is normal and desirable for **unplanned events to influence** interests, attitudes, preferences, etc. | Warner’s decision to dump Elle caught her by surprise and was very painful for her, but that setback is what led to her exploring law. She would never have become a lawyer had she realized her initial plan to be Warner’s wife. | Tony Stark was not planning on getting abducted by insurgents when he visited the Middle East. However, he used that situation to build his first iron suit.. |
| It is worth the effort to **promote and take advantage of happenstance** situations. | Elle studied hard for the LSAT, but she also took advantage of her good looks and used a video essay for her Harvard application. | Tony Stark took advantage of the technology he developed for the unplanned need to keep shrapnel away from his heart and, using that technology, worked tirelessly to create The Iron Man. |
| **Skills** |  |  |
| **Curiosity:**  Exploring new learning opportunities | When Elle was feeling upset and lonely at Harvard, she explored opportunities to connect with others at multiple places and found friends at the nail salon. | Tony Stark was constantly looking for ways to improve his Iron Man suit. |
| **Flexibility:**  Changing attitudes and circumstances | Initially Elle’s goal to attend Harvard was to win back Warner, but she changed her goal when she realized he would never a value her. | Tony Stark was flexible in his thinking when he changed his attitude about his role in arms distribution. He had to completely rethink his role and his contribution to society. |
| **Risk-Taking:**  Taking action in the face of uncertain outcomes | Elle accepted the challenge to be Ms. Brooke Taylor’s lawyer, even though she was only a law student. | Tony choose to completely stop weapons development (which was what his company made the majority of their money on) because he wanted to rewrite his legacy in more impactful way. |
| **Optimism:**  Viewing new opportunities as possible and attainable | Elle was optimistic about her ability to get in to Harvard even though she didn’t fit the mold. “It’s not like it’s hard or anything.” | Tony Stark was very optimistic he could create something that had never been created before. He was also optimistic that Pepper Pots would handle his company well, freeing his mind and time for creating the Iron Man. |
| **Persistence:**  Exerting effort across time, even despite setbacks | Elle was not discouraged by the doubts of her parents, professors, or classmates. She worked hard to get into Harvard and to prove her spot there. | Tony’s arsenal of suits got destroyed how many times? He’s saved the world how many times? Yet he keeps stepping up, donning another suit, and saving the world. |

| **Attitudes** | **Ben’s Story** | **Your Story** |
| --- | --- | --- |
| Indecision is not a problem to be fixed, but **a “planful” open-mindedness** to future possibilities. | Even though Ben was interested in medicine, he did Teach for America right out of college, which turned out to be “a pivotal moment” |  |
| It is normal and desirable for **unplanned events to influence** interests, attitudes, preferences, etc. | When Ben agreed to do Teach for America, he didn’t know he would be assigned to work with adolescents. Yet, in the end, it reinforced his interest in pediatrics. |  |
| It is worth the effort to **promote and take advantage of happenstance** situations. | Ben took advantage of his mom to work in the NICU to “get his ducks in order” to apply to med school, doing so led to realize that working with kids “seems right” |  |
| **Skills** |  |  |
| **Curiosity:**  Exploring new learning opportunities | “After teaching for a few years, I got into medical school. I knew that I wanted to do work with teenagers, I was … flirting with other specialties” |  |
| **Flexibility:**  Changing attitudes and circumstances | “I remember this baby who was freaking out and thinking to myself. “oh, my gosh, this is NOT what I want to do.” The second day though, I had an amazing teenager … My mindset changed.” |  |
| **Risk-Taking:**  Taking action in the face of uncertain outcomes | “A pivotal moment in my career was after college when I taught for Teach for America in a public middle school... It was hard work, but it felt great. |  |
| **Optimism:**  Viewing new opportunities as possible and attainable | “I taught sixth grade and they were at a vulnerable age, fun and impressionable age. I liked that they were ornery, and naughty and starving for information.” |  |
| **Persistence:**  Exerting effort across time, even despite setbacks | “I think I didn’t know it then but finally arriving at it years later, what I care most about” is working with marginalized youth. |  |
