# Supplementary material for: Planning for Happenstance: Helping Students Optimize Unexpected Career Developments
Source: MedEdPORTAL. 2021 Feb 8;17:11087. doi: 10.15766/mep_2374-8265.11087 (PMC7880249; doi:10.15766/mep_2374-8265.11087)
Supplement: Supplementary file 1 — Eight Stories.docInstructor Guide.docPowerPoint Slides.pptxJason's Story PHLT Video.mp4PHLT Worksheet.docxPHLT Workshop Evaluation.docx [file mep_2374-8265.11087-s001.zip › F. PHLT Workshop Evaluation.docx]

Planning for Happenstance:
Helping students optimize the unexpected as part of their professional development

What is your current position/role in medical education? (check 1)

- Student
- Resident
- Staff
- Faculty
- Other

1. Think back to before this workshop. Please rate your agreement with the following statements from your perspective **BEFORE** the workshop

|  | Strongly Strongly  Disagree Disagree Neutral Agree Agree |
| --- | --- |
| Indecision is not a problem to be fixed, but a planful, open-mindedness to future possibilities. | 🔾 🔾 🔾 🔾 🔾 |
| It is normal and desirable for unplanned events to influence interests, attitudes, and preferences. | 🔾 🔾 🔾 🔾 🔾 |
| It is worth the effort to promote and take advantage of happenstance situations. | 🔾 🔾 🔾 🔾 🔾 |

1. Now the workshop is over. Please rate your agreement with the following statements from your perspective **AFTER** the workshop.

|  | Strongly Strongly  Disagree Disagree Neutral Agree Agree |
| --- | --- |
| Indecision is not a problem to be fixed, but a planful, open-mindedness to future possibilities. | 🔾 🔾 🔾 🔾 🔾 |
| It is normal and desirable for unplanned events to influence interests, attitudes, and preferences. | 🔾 🔾 🔾 🔾 🔾 |
| It is worth the effort to promote and take advantage of happenstance situations. | 🔾 🔾 🔾 🔾 🔾 |

1. In your opinion, how important/relevant are the planned happenstance skills to career decision making?

|  | Not  Important Neutral Important |
| --- | --- |
| Curiosity: Exploring new learning opportunities | - ➁ ➂ |
| Flexibility: Changing attitudes and circumstance | - ➁ ➂ |
| Risk-Taking: Taking action in the face of uncertain outcomes | - ➁ ➂ |
| Optimism: Viewing new opportunities as possible and attainable | - ➁ ➂ |
| Persistence: Exerting effort across time, even despite setbacks | - ➁ ➂ |

1. How confident are you in advising/coaching students/peers to develop planned happenstance skills?

|  | Very Very  Unconfident Unconfident Neutral Confident Confident |
| --- | --- |
| Curiosity: Exploring new learning opportunities | - ➁ ➂ ➃ ➄ |
| Flexibility: Changing attitudes and circumstance | - ➁ ➂ ➃ ➄ |
| Risk-Taking: Taking action in the face of uncertain outcomes | - ➁ ➂ ➃ ➄ |
| Optimism: Viewing new opportunities as possible and attainable | - ➁ ➂ ➃ ➄ |
| Persistence: Exerting effort across time, even despite setbacks | - ➁ ➂ ➃ ➄ |

1. Now think about planned happenstance in your own career. Which of the following skill(s) would you most like to strengthen in yourself to enhance your ongoing career decision-making? (check all that apply)
   - Curiosity: Exploring new learning opportunities
   - Flexibility: Changing attitudes and circumstance
   - Risk-Taking: Taking action in the face of uncertain outcomes
   - Optimism: Viewing new opportunities as possible and attainable
   - Persistence: Exerting effort across time, even despite setbacks

*What are 2-3 steps you can take to realize that goal?*

|  |
| --- |

1. Please give us your feedback about the strengths and areas for improvement for the workshop. Thank you.

- Strengths:
- Areas for improvement:
