# Supplementary material for: Planning for Happenstance: Helping Students Optimize Unexpected Career Developments
Source: MedEdPORTAL. 2021 Feb 8;17:11087. doi: 10.15766/mep_2374-8265.11087 (PMC7880249; doi:10.15766/mep_2374-8265.11087)
Supplement: Supplementary file 1 — Eight Stories.docInstructor Guide.docPowerPoint Slides.pptxJason's Story PHLT Video.mp4PHLT Worksheet.docxPHLT Workshop Evaluation.docx [file mep_2374-8265.11087-s001.zip › A. Eight Stories.doc]

Appendix A:

**Stories of Happenstance**

This document contains eight unique accounts of how happenstance influenced the career trajectories of young, but established physicians.

.

In the Planned Happenstance Workshop you will only have time to use 1 or 2 stories. Pick the story(ies) which you feel will best appeal to your participants.

The stories were made possible by funding from the Associate of American Medical Colleges, Group on Educational Affairs (AAMC-GEA) as part of a research initiative to explore physician career inflection points

# Ben’s Story

## Synopsis

### For Ben, teaching was a ‘pivotal moment’ leading to a satisfying career in adolescent medicine, because manhandling a baby wasn’t rewarding.

## Summary

### “I think when I was younger; I didn’t know what I wanted to do. I think people imagine medicine as this calling, and for me, it wasn’t a calling. I never thought that’s what I was going to be. I was a bright kid. Not brilliant, but I was bright. My mom was a nurse, so we had scrubs and a stethoscope in the house, I would be a doctor for Halloween I went to college as someone planning to go to medical school. This meant you had to have your ducks in a row and do some volunteerism. My volunteer work was a cuddler in the NICU. Around this time, a lot of research came up showing how human contact was important for the survival of the little babies. Once a week I would walk into the NICU, gown up and hold babies for an hour. I remember sitting in the chair, rocking a baby, and thinking how amazing this was, with all of the beeps and movement and bustle of the NICU. Hospitals and NICUs, they have smells and, noises that can turn people off. But I had no aversion to it; It was- more like, I like this environment, this space it wasn’t an aha moment, or. “0h, my god, this is what I have to do.” But it just was like, okay, I can do this, this seems right.

### A pivotal moment in my career was after college when I taught for Teach for America in a public middle school. That is when I fell in love with adolescents. I taught sixth grade and they were at a vulnerable age, fun and impressionable age. I liked that they were ornery, and naughty and starving for information. I loved that they were critically appraising everything around them at all times. That was when I was, okay, I have to do this. I have to work with this age group forever. It was hard work, but it felt great. I really enjoyed going to work.

### After teaching for a few years, I got into medical school. I knew that I wanted to do work with teenagers, I was tinkering with subjects, flirting with other specialties. I had a really good mentor who was a surgeon. I remember going the first day of pediatric clerkship and it was a nightmare. It was basically all these well-child-checks I remember manhandling this baby who was, freaking out and thinking to myself. “oh, my gosh, this is NOT what I want to do.” The second day though, I was in adolescent clinic and I had an amazing teenager who had the deck stacked against her and was just super marginalized, just impoverished, and running away from home and was abused, a lot of trauma— but was so resilient. She had a positive attitude despite everything being against her. That 40 minutes I got to spend with her was just so powerful. My mindset changed. I was like, “Okay, I can do pediatrics because it gets me to adolescents.” Now, having done 5 years of adolescent medicine and working with marginalized youth, that’s when it feels like now the right calling. I think that I didn’t know it then but finally arriving at it years later, this is what I care most about.

## What we learn

### Every physician’s story of their journey into medicine is unique. Factors, like family, personal interests, relationships, give rise to unforeseen opportunities and decision points. They become stepping-stones on a path to choosing a specialty. Many of these factors seem random or based on luck. But are they? Let’s revisit Ben’s story.

### If Ben’s mother wasn’t a nurse, Ben would not have had scrubs to wear for Halloween and Ben might not have volunteered as a cuddler in the NICU. So, in a sense, Ben was lucky to be born to a mother who was a nurse. Similarly, if Ben had been assigned to a different age group of students with Teach for America, he might not have discovered how much he loved working with kids who were “ornery and naughty and starving for information.”

### So luck does seem to play a role. But we think there’s more to the story. We think the impact of these seemly random events, was heavily shaped by Ben’s attitudes and actions—things that go way beyond luck. Ben was actually more in control of his journey then he even realized. Let us explain.

### Ben showed other important intrinsic traits that are important to develop. For example,

#### Ben had a propensity for reflection, to be mindful of what was happening and putting it into the larger context of his goals and values.

#### As a student, Ben avoided jumping to conclusions after a first bad day on pediatrics. Instead, he gave it more time only to learn how wrong his decision would have been to have excluded pediatrics as a specialty of interest.

#### Ben explored options but held onto his intrinsic priority of working where he’d have real human connections.

#### And finally, Ben turned Indecision into planfull openness and was willing to stay for a time with that indecision and not jump prematurely into decisions he’d later regret.

# Jennifer’s Story

## Synopsis

### Jennifer never followed the default path in becoming a physician. The decision for ‘deliberate diversion’ gave room for explorations within her medical career.

## Summary

### I’ve never liked following the default path, but it’s easy to do that in medicine, to just chug along because the system pushes you along. Or you think it’s the best thing to do, the best place to go because it has the best reputation, instead of finding what will work best for you.

### Having great mentors also influenced many of my choices. I came into medical school always thinking I would do pediatrics. I didn’t even think about internal medicine until my fourth year when I had a great pulmonary attending who tried to convince me to do just medicine. I knew I was a pediatrician at heart but I decided to try both and started a med-peds residency. In the end, I went into a pediatric subspecialty but I’m glad I tried med-peds. Like I said, I don’t like to follow the default path. I’ve taken several deliberate diversions in order to experience life the way that I want to. Taking these types of diversions meant I have had several gaps in my medical training that I’ve had to explain. I worried a bit about that, but in the end, I think those experiences informed my choices and I would not have done it any other way.

### For example, I met my husband in medical school and he had a goal to work overseas. We decided to take a year off to work in Nepal for a year. For him, it was at the end of residency but for me, it was in the middle of residency. We worked it out though because it felt important for both of us to do global health before we settled our careers and started a family.

### I took another ‘deliberate diversion’ between residency and fellowship when my son was born. I had my few years later when I was a fellow. Having two babies during training was really exhausting. So, I decided to work part-time after I graduated. I got to be home with my little children, but it also helped me realize how much I enjoyed being a working mom.

### We recently picked up and moved to another state so we could be closer to family. My husband and I wanted our kids to be closer to cousins and grandparents. Looking back, I think these kinds of deliberate diversions help you realize the choices you are making consciously rather than just going along the path that you are expected to go on.

## What we learn

### *Jennifer showed flexibility and adaptability*. When she started medical school she wasn’t planning on going to Nepal or having two children; but by thinking creatively and persisting in her plan to become a doctor, she was able to finish medical school and have these experiences.

### *Jennifer experiences were influenced by ‘happenstance’ as well*. Her decision to work her medical career around her young family worked, in part, because she happened to be in a work environment that supported her. Also, her interest in meds began from a teacher she happened to take a course from in her fourth year.

### *Jennifer’s* Career has been shaped by both chance and choices she made

# Sarah’s Story

## Synopsis

### Identified as an “athlete”, Sarah used her sports experience to persist and tackle challenges within medical training. Ultimately, finding the right ‘fit’ for her.

## Summary

### When I was in college, I did well academically, but I didn’t push myself. My focus was on playing basketball. I was very dedicated to playing my sport: “athlete” was my identity. I even played professional basketball for a while. But after getting injured a few times, I started looking for a new career—something that would challenge me mentally, just as basketball had challenged me physically. I think I could have been happy choosing a number of things.

### I think I ended up choosing medicine because of my family. My parents stressed the importance of having a career where I could support myself, especially as a woman. Medicine offered job security, a decent salary, and would be very challenging. This is why three of my older sisters went into medicine. I watched them go through medical school and knew it could be tough, but worth it. Also, I had a niece who had a form of hypoplastic left heart and lived for only 3 weeks. I spent time with her at the PICU and I saw how her illness impacted my family.

### Looking back, I think my basketball career prepared me for medical school because I got used to being tough. I experienced people yelling at me, working through exhaustion, some people not seeing me as an individual. I also learned sometimes life is just unfair; sometimes you work really hard and still don’t get what you want. But, I learned not to take any of it personally and to move on. Sports also taught me to not second guess my decisions.

### During medical school, I spent a month in Boston doing neonatal neurology. My peers thought it was probably not worth all the effort and discouraged me from going. Turns out it was totally worth the effort, especially later in my residency when it helped me make future career decisions. And I met a mentor who I still keep in touch with. Not only can I call her whenever I need, we meet up once a year at our professional society meeting. She has helped me build a world-class department here at the University.

### But I didn’t know that is where I would end up when I was in medical school. I knew I didn’t enjoy working with adults, so I went into pediatrics. But, I had a feeling I didn’t want to do general pediatrics, like my sisters. I started my residency not sure which pediatric subspecialty would interest me. I liked neurology but didn’t know there was a peds neurology path. When I found out about a peds neurology elective, people told me nobody does that because the hours are long, However, once again, I decided to try out something that seemed interesting to me, despite the challenges. It wasn’t until I was doing that, that I knew I had found the right ‘fit’.

### I came to the U because my family wanted to move and we like hiking and wanted somewhere warmer. Also, I met Dr. F. and very quickly knew I wanted to work with someone like him. Turned out the U was great at allowing people to take their area of interest and run with it. I’ve had to make my own path at times, but it’s been a fantastic experience with wonderful mentors along the way. The challenges have all been worth it in the long run.

## What we learn

### Unplanned life experiences can create positive learning opportunities with optimism. Sarah applied some of the life skills she learned from basketball to being a medical student (e.g., coping with unexpected events such as injuries or emotionally challenging team conflicts). She learned persistence pays off…up to a point. While she did not quit after her first injury, she eventually realized her body could only handle so many injuries and that continuing to play professional basketball would have long-term negative consequences. Deciding to go into medicine was a big risk.

### Openness to different experiences in medical school can prepare us for uncertain futures. Sarah was willing to take risks in her medical schooling. She was not sure exactly what she would do in pediatrics, but it felt right to her, so she ‘jumped in’.

### She manifested curiosity and did some risk taking on challenging electives and a rotation in Boston despite being discouraged to do so by colleagues. In the end, these experiences were very positive and influential in her career. In other words, she was willing to sit with uncertainty and try things out.

### Success is a team sport. Sarah took advantage of serendipitous experiences with others, forming relationships with individuals that helped shape her career trajectory. Early on, she could not help being curious about her sisters’ experiences and learned from them both, positive and negative.

### Extreme risk and flexibility for Sarah She went to Boston for a rotation with little knowledge of what to expect. In the end, her trip there enabled her to cultivate a long-term relationship with a mentor whose influence continued well beyond the few weeks she was there and shaped her career in fellowship, many years later These long-lasting relationships provided a ‘safe space’ to explore options and get feedback and advice.

# Taylor’s Story

## Synopsis

### Taylor is a community advocate who worked to maintain empathy in residency and stay true to herself.

## Summary

### My first years in medical school felt a lot like drinking from a fire hose. I felt I had so much to learn, there wasn’t a lot of time to reflect. The most I could say was “I know I’m in the right profession.” In my third year, I struggled because I felt I should go into surgery. I could pick it for a lot of good reasons, but at the end of the day, I couldn’t say I loved it for me.

### In my fourth year, during a rotation on abuse, I worked with a doctor who specialized in best practices for sexually abused children. I saw a little girl reach up and slap the doctor while she was measuring the child’s bruises. I remember thinking that this child was simply modeling behavior. I felt strongly that we, as adults, caregivers, and a society, had led to this outcome for her. At that moment, I realized the community work that I had done as an undergraduate was still really important to me and here was an opportunity for me to engage in ongoing advocacy.

### It still took me a little while to commit to pediatrics, because my partner was concerned I wouldn’t be making as much money as I could in other specialties. However, I knew it was in keeping with my value system and felt strongly we would be just fine. I felt like if I was going to dedicate my life to others, then it better be doing something I feel passionate about.

### When preparing for residency, person helping me match was quite adamant that I would not like going to N. I worried that there was something I didn’t understand because I didn’t see a problem with going to N. After interviewing several places, it seemed like N was going to give me the best opportunities to work with different types of people. That was really important to me. So, I followed my intuition and went to N. This experience seared in my mind two things. One, there will be times I’ll think “there’s something I don’t understand here, but I feel right about this and I’m going to learn from it”. Two, it’s okay to say “I think I know myself and my needs better than this person that I'm supposed to have advise me.”

### I think residency training can erode our empathy. Three things helped me during those tough years. One the owner of a popular deli always remembered our order, not always our name, but he remembered what we like to eat. He taught me there are many ways humans can connect with one another. Two, I followed the advice of my mentor and spent time at the local juvenile justice center and watched attorneys advocate for children. I noticed a high variability in their fight for their clients. I couldn’t believe a child's future could be in the hands of somebody who might not actually care. I decided I didn’t have to agree with parts of their lives, but I would always care about my patients. Third, after a rotation, I’d often go to a local art museum. I’d be really tired, but seeing the sculptures and paintings reminded me of the centuries old mission and beauty of my profession.

## What we learn

### *Values matter*: For Taylor, staying true to her values and finding ways to reconnect with her values were an important part of her success during her medical training. Whether making the decision to go into pediatrics, or learning from deli owners, doctors, or community museums, staying in tune with her values helped her recognize important growth opportunities.

### *Flexibility matters*: Taylor allowed herself the space she needed in medical school to settle on a specialty. She kept herself open to possibilities and thus was able to recognize when the ‘happenstance’ moment of working with Dr. F. could lead to a successful career.

### *Advocacy matters*: For Taylor, social advocacy was important. But we also see from her the importance of self-advocacy. She had the courage to attend a school that wasn’t recommended to her and to choose a career other than surgery because she believed in caring for herself and her own needs.

# Nebraska’s Story

## Synopsis

### Medicine is a combination of science, people, and research. With the guidance of mentors, Nebraska stayed adaptable during setbacks sequentially creating room for evolving new opportunities without limitations.

## Summary

### I think I went to med school because that’s what smart people did. My undergraduate degree is in biological systems engineering. My first summer in college I took a job doing bench research. I had not sought out research per se, I just needed money to stay in the area to take summer classes. I found the job flipping through an employment binder at the science building. I worked there for 3 years and discovered I loved doing research. But, I couldn’t imagine myself in a lab permanently because I like people. Medicine was science and people, with some research involved.

### I didn’t know I liked being around children until my second year in med school. I went to a pediatrics infectious disease lecture with some friends just because I wanted the free lunch. I ended up really enjoying the lecture so I talked to the presenter afterwards. She agreed to let me shadow her and introduced me to Dr. S who invited me to work in her peds ID lab. Then in my third year, the highlight of my day was seeing the kindergarten physicals. I didn’t expect that, but the kids were so happy and hilarious and I couldn’t help but have fun.

### After my general pediatrics residency, I interviewed for ID fellowships all over the country but felt like I wouldn’t find a better mentor than Dr. S and I knew she’d give me the flexibility to pursue another path if six months later I was tired of research.

### One day, during my fellowship, several of us were at a coffee shop and chatting about the unusually high number of shunt infections we’d seen. We started brainstorming ideas better ways to do it. I took those ideas, researched them, and pursued a few. That’s what brought me to where I am now.

### I think two personality traits have most influenced my career. One is being introverted; while-I like people, I also know that seeing 40 patients a day would be extremely exhausting, so I focused on research. The other is being really stubborn; in research, it’s easy to give up when a technique that you thought was going be fantastic turns out to be a huge bust. You have to be adaptable and say this is going terribly, now what am I going to do?

## What we learn

### Curiosity to explore new opportunities in shaped Nebraska’s happenstance; however, she took actions that increased in her ‘luck’ in these situations. For example, she chose the science building binder and made cold calls to find a job. She approached the lecturer about learning more on a topic that piqued her interest. Finally, she used a coffee break conversation as a foundation for research that leads to her career. In other words, she took advantage of seemingly small events and used them to her advantage.

### Proactive Mentorship was an important influence throughout Nebraska’s career. The unplanned event for the person to allow Nebraska to shadow her, which led to other influential connections, helped her career trajectory.

# Evan’s Story

## Synopsis

### Exposure within the hospital environment as a child blossomed into a career in pediatric medicine. Although, Evan didn’t make it the first attempt into medical school, he stayed optimistic to persist and eventually was accepted.

## Summary

### My older sister has spina bifida. When I was in eighth grade she was frequently in the hospital. I spent a lot of time there, hanging out in the game room and playing video games with the pediatric patients. I think my interest in pediatrics started there.

### In high school I had in mind I would go to medical school, but I didn’t work hard enough college to get into medical school. I spent a couple of years retaking some course and building up my GPA. However, I still wasn’t accepted into medical school. I decided to try one more time, working harder and smarter. But I also formulated a backup plan: do a Ph.D. in biology and stay within academics.

### When I made it into medical school on my second try, I remained focus on doing well in class. I attended pre-clinical lectures regularly, even when they took place early in the morning. I found that the pediatric hematology/oncology lectures grabbed me—it thinks it was something about the content as well the lecturer. During my 4th year, I took an elective from him and became convinced that he was the one I most wanted to be like as a doctor—a great combination of humor and passion.

### Aside from my experiences in medical school, I was attracted to hematology/oncology because of my girlfriend. We met at a summer camp for diabetic kids, where I had volunteered. During the time we saw each other, she developed melanoma. Sometime after we broke up, I learned that she had died. Upon reflection, her death did not lead me into oncology but drove me to stay in oncology. It also helped me be more empathetic with my patients and their families.

### In fact, one of the things I like most about hematology/oncology are the families I work with. They inspire me with their resilience and a strong determination. I also enjoy the chance to treat once healthy children who are now sick and make them healthy again. (Instead of the adult who says “I kind of did this to myself, so now please take care of me, just so I can revert back to my old habits”.) These children inspire me to work harder, rather then frustrate me with their habits.

## What we learn

### Optimistic and Flexibility to different experiences are important. He had to exposure to new surroundings while his sister’s illness was a difficult time for everyone, he learned from the experience. Including the unfortunate passing of his ex-girlfriend, which is a seed for his reassurance in HemOnche.

### Persistence to retaking classes, work smarter, work harder to achieve his goal of starting medical school.

### Curiosity to explore different lectures, he might not have had the opportunity to be inspired by a role model and the interest in hematology/oncology.

# Johnathan’s Story

## Synopsis

### Johnathan grew up hearing about hospital patients, but it was treating a patient with constipation that shaped his career. He persevered through a murky residency to fulfill his dream of developing a pediatric palliative care program.

## Summary

### My dad is an adult intensivist, my mother is a social worker, and at dinner, they often discussed how to support their patients and families. I was surprised later to find out my dad did more as a doctor then “just talk to people.” He explained that it was his favorite part of being a doctor. As a child, I did not want to grow up to be a doctor, but my dad’s lesson about what is most important in healthcare really stuck with me.

### As a kid, I was scared of hospitals. I would not go in one, not even to see my grandparents. However, in college I kept coming back to the idea since I liked science and working with people, it made sense to be a doctor Therefore, I decided to spend a summer interning at the hospital where my dad worked. No one thought I would ever become a doctor because of my fears, but I overcame that obstacle by spending time in the hospital every day. I ultimately loved being there.

### In medical school, I wanted to feel useful. One time, a COPD patient mentioned that his biggest problem was chronic constipation. I advocated hard with the medical team to do something for him about this. At first, they only wanted to treat his COPD. The next morning, the patient gave me a big grin and said: “I just took the best dump of my life.” Crazy as it sounds, that was a pivotal moment for me. I had helped someone experience better care and it felt important to me. When I took an elective in palliative care, the team let me take an active role in working with patients. I decided palliative care was one way I could really make a difference in people’s lives.

### Things got a bit murky in residency. I loved the peds rotation but no one was doing pediatric palliative care at my school. I sought out a month’s rotation at B- Children’s Palliative Care Team, but most of the kids were nonverbal and I missed having meaningful conversations with patients. Therefore, I switched to hospital medicine and got a fellowship. After that, I did a palliative care fellowship in O-. Eventually, I returned to B-, where I did my meds fellowship because my fiancée was still there. That is when I met Dr. Q, who offered to support me in developing a pediatric palliative care program. For a while, I did both—palliative and hospital medicine. However, just recently I made the decision to do pediatrics palliative care full time.

## What we learn

### It was an *unplanned happenstance* his father and mother both work with patients and brought their work life into the home. This exposure for Johnathan may have been a subconscious inculcation into medical school.

### There was an element of *risk taking* to start shadowing in the hospital. Because Johnathan was deathly afraid of hospitals.

### *Curiosity* and *flexibility became key for Johnathan* in exploring palliative care. Although he originally planned to become an ER physician.

### Ultimately, *Persistence* to do palliative care was evident because he had played with different ideas during his residency but always fell back to palliative care. This is visible during the COPD rotation.

# Ian’s Story

## Synopsis:

### Ian’s family's pediatrician introduced him to a career in medicine.  A 'perfect storm' of negative events changed his direction for the better.

## Summary

### Medicine is a very glorified thing, especially on TV - quite different than what I see in my dad (a farmer) and my mom (a banker). When I told my pediatrician, who was also a family friend, I was interested in being a doctor, she invited me to lunch and talked about the real-life experiences of being a physician. She told me, ‘If you remember nothing else from this, remember medicine is a great privilege and a great burden. If you’re willing to take on the burden then we can talk about the privilege’. She got me in touch with people who gave me clinical research experiences in pediatric critical care. This exposure really helped me get into medical school because my MCAT scores were only so-so, but I had a really strong CV.

### My interest in peds happened early and stayed with me. One time, while volunteering in pediatric critical care, I got to go with the transplant team. I was carrying the organ cooler in the elevator when a patient’s mom got in. When she realized her son was getting the transplant, she began to cry and hug us. Seeing her relief and thankfulness really shaped my decision to be a pediatrician; no one is going to be as happy as a parent will be for their children.

### During medical school, I joined the Ethics Committee. I was a philosophy minor in college and really enjoy ethics. So when I met Dr. Y, who focused on ethics in intensive care, I felt that intensive care was the right place for me. Watching her successfully fight for what she thought was right was inspiring and gave me courage to act on what I believe is right.

### In my fourth year, a child had an intra-operative complication and the attending, Dr. X, insisted we diurese him. I felt like it wasn’t the right thing to do. Others, including senior staff, agreed with me but said we had to do what he wanted because of his position, regardless of how it affected the patient. I felt I couldn’t work in this kind of environment.

### I had a bit of meltdown at the thought of changing paths in the middle of my fourth year. Eventually, I realized I liked working with children who have pulmonary hypertension. Although no one had ever changed from critical care to pulmonary, I had a mentor who believed in me helped me. When I told my partner about my plan, he said “I‘m so glad, 'cause you’re always so miserable when you’re out there [in ICU]” As much as I hated that interaction between Dr. X and how I felt at the time, it was a perfect storm that helped me realize what I thought I wanted wasn’t really for me. I think I would have ended being miserable if I had stayed.

## What we learn

### Unplanned events become lucky opportunities. Ian was lucky enough to have a pediatrician who looked out for him. But he made the choice to sacrifice some of his time to try out shadowing different doctors.

### Curiosity, Lead to research opportunities, ultimately guiding him into the transplant unit.

### Persistence to continue being part of the transplant unit kept him in the direction of CVICU.

### Sometimes risk taking is necessary after an event especially for Ian after his experience with Dr. X1, he changed his trajectory to other focus in pediatrics.

### Ian stayed flexible and optimistic, using his background in ethics to influenced him and build a foundation around it.
