# Supplementary material for: Planning for Happenstance: Helping Students Optimize Unexpected Career Developments
Source: MedEdPORTAL. 2021 Feb 8;17:11087. doi: 10.15766/mep_2374-8265.11087 (PMC7880249; doi:10.15766/mep_2374-8265.11087)
Supplement: Supplementary file 1 — Eight Stories.docInstructor Guide.docPowerPoint Slides.pptxJason's Story PHLT Video.mp4PHLT Worksheet.docxPHLT Workshop Evaluation.docx [file mep_2374-8265.11087-s001.zip › B. Instructor Guide.doc]

Appendix B: Facilitator Guide

**Introducing Concepts of**

**Planned Happenstance Learning Theory
 to Mentors and their Learners**

# 1. Precis

## 1.1. This document is a guide for introducing Planned Happenstance Learning Theory (PHLT) to Learners and their Mentors. It describes resources prepared and piloted with funding from the GEA that facilitators can use in PHLT workshops for mentors.

## 1.2. The document begins with background information and learning objectives and then includes detailed explanations of and instructions for using available resources.

# 2. Background

## 2.1. Boyd Richards and Dorene Balmer and their colleagues obtained funding from the AAMC GEA to explore career inflections points with faculty from four medical schools. They recorded and transcribed interviews with 6 faculty from each institution. In these interviews, they asked the faculty to identify and discuss their career inflection points (events that altered the trajectory of the path leading to their current career).

## 2.2. In analyzing these transcripts Richards et, al., found that a large number of faculty referred to ‘luck’ or unexpected moments in describing their inflection points (between 5-8 per interview). When they went to the literature to see if similar findings have been reported elsewhere, they encountered the framework of Planned Happenstance Learning Theory (PHLT)[1]. Intrigued, they used PHLT as an interpretive lens of their interview data and found it very enlightening and helpful. The concepts central to the PHLT framework resonated with their overall impressions of the interviews as well as their personal views on career development. As a result, they selected two interviews per school and closely read the transcripts looking for examples of PHLT at play. By the time they were done, they and captured the essence of these 8 interviews in simple narratives with accompanying notes (see Appendix A).

## 2.3. While there won’t be time to review all the stories in the workshop, we include them in the appendix to help you become familiar with PHLT and how it was manifest in the careers of these individuals. We suggest you select one or two stories to include in the workshop (i.e. Ben).

# 3. Workshop Learning Objectives

## 1. Recognize the importance of Planned Happenstance Learning Theory **attitudes** in career development

## 2. Recognize the importance of Planned Happenstance **skills** in career development

## 3. Express confidence in **advising/coaching students** and/or peers in use of Planned Happpenstance Learning Theory attitudes and skills in learners’ career development

# 4. Contents

## 4.1. PPT presentation with identified locations for application activities (see Appendix C)

## 4.2. Digital story from 4^th^-year student about his planning for and learning from happenstance (see Appendix D)

## 4.3. PHLT worksheet, two sided (see Appendix E)

### 4.3.1. Side 1—for use in workshops with 75 minutes or more: PHLT attitudes and skills illustrated with PopCulture (Legally Blond and Iron Man)

### 4.3.2. Side 2: PHLT attitudes and illustrated with Ben’s story and with blanks for participant’s to use to make notes about their own personal story

## 4.4. 3-part workshop evaluation

# 5. Workshop Preparation

## 5.1. Select one or more of the stories of happenstance included in Appendix A. We recommend Ben’s story, but any will work.

## 5.2. Make sufficient copies of your selected story(ies) so that each participant as their own copy.

## 5.3. Make sufficient copies of the PHLT Worksheet (Appendix E).

## 5.4. Decide whether you want to conduct your evaluation with paper immediately after the workshop; if so, make a sufficient number of copies.

## 5.5. Consider if you want to use flipcharts and if so, acquire them.

# 6. Workshop Timeline

| **Duration** | **Content** | **Slide Numbers** |
| --- | --- | --- |
| 5 min | Welcome; Introductions | 1-3 |
| 15 min | Background of Research | 5-7 |
| 10 min | Stories and discussion | 8-11 |
| 10 min | Introduction to PHLT | 12-16 |
| 15 min | Learning from Ben Story | 17-18 |
| 15 min | Self-Reflection | 19-23 |
| 10 min | Helping Students apply PHLT | 24 |
| 10 min | Wrap up & Evaluation | 25 |

# 7. Workshop Flow

## 7.1. The facilitator uses this Instructor Guide and PPT slides (see Appendix C) to guide the flow of the workshop beginning with introductions and a brief review of the objectives and agenda.

## 7.2. After a brief introduction to the interviews which gave rise to our interest in luck, the facilitator introduces the idea of planning for luck, first with a quote from Thomas Jefferson and then with a brief video testimonial from a fourth-year medical student (see Appendix D).

## 7.3. With participants primed to consider the value of planning for luck, the facilitator introduces the attitudes and skills central to Planned Happenstance Learning Theory[1, 2] using PPT slides.

## 7.4. Time permitting (in workshops with more than 75 minutes), the facilitator illustrates application of the attitudes and skills of PHLT using reference to two movies from pop culture, Legally Blonde and Iron Man.

## 7.5. As a transition to helping workshop participants reflect on their own use of PHLT attitudes and skills in their professional planning, the facilitator asks participants to read the story of Ben, one of the eight vignettes (see Appendix A) captured from faculty interviews of career inflection points.

## 7.6. The facilitator also asks participants to review the summary provided of how Ben manifested PHLT attitudes and skills using the same worksheet participants will be using to reflect on happenstance in their own experiences (see Appendix E).

## 7.7. After a brief discussion, the facilitator asks participants to use the same worksheet to reflect and make notes about their personal journeys with happenstance before sharing their notes with a peer, using a traditional think-pair-share format. Facilitators circulate among the participants to answer questions, monitor progress and refocus if necessary.

## 7.8. This activity is followed by a large group discussion.

## 7.9. The workshop ends with a guided discussion of ways to introduce PHLT to learners and ways to promote PHLT attitudes and skills when advising and mentoring students about their ongoing career development.

## 7.10. In the final 5 minutes of the workshop, facilitators ask participants to complete anonymously a brief internally-developed workshop evaluation (see Appendix F), the results of which can be used to make improvements in content and organization of the workshop materials. This evaluation consists of three parts: 1) questions soliciting participant feedback about the content and organization of the workshop, 2) questions asking participants to indicate their level of agreement with statements about the relevance of PHLT attitudes and skills in career development, both prior to and after the workshop (using a retrospective pre-post approach), and 3) questions asking participants to indicate where they are most committed to change some aspect of their practice involving application of PHLT attitudes and skills.

# 8. Facilitator Notes by Slide

| # | Slide Contents |  |
| --- | --- | --- |
| 1 | Title Slide: | Welcome participants |
| 2 | Disclosures | If you think it will make a difference with your audience, you could identify the schools at this point: University of Utah, University of Pennsylvania/Children’s Hospital of Philadelphia, University of Nebraska, Baylor College of Medicine, University of Mississippi |
| 3 | Objectives | These objectives assume that most participants will not be familiar with PHLT, so the workshop first introduces PHLT and then explores how to help learners use PHTL |
| 4 |  | As you think time and participant interest will allow, briefly summarize multi-institutional research that gave rise to the interest in planning for happenstance. (See instructor guide for more details of this research.) |
| 5 | “I'm a greater believer in luck, and I find the harder I work, the more I have of it.” | Read the quote, pause so that participants have a moment to reflect, and then explain that the quote supports recognizing the role of “luck” or happenstance in our lives. |
| 6 | Planning for Happenstance – Luck is No Accident | One way to think about planning for happenstance is to recognize that “luck is no accident”. It is interesting to note that the authors of the PHLT (Krumboltz and Levin), use this title a book they wrote to share the specifics of the theory in a practical way. |
| 7 | ? | We include slides with questions to serve as a pause, and chance to see if participants have any questions. Also to give a look ahead about what’s next. You do not need to read the text for what is coming up next. |
| 8 | A Student’s Story | Use this slide to prepare participants to view the video with Jason’s story (Appendix D) so that it has a greater impact. The video is intended to pique interest in the attitudes and skills of Planned Happenstance Learning Theory.  The story was recorded by a student in his fourth year of medical school. It describes some of the moments of happenstance that redirected his career decision-making and how he responded. |
| 9 | MP4 of story | This is where the video of a student testimonial about happenstance in career development can be shown. (Appendix D). **In advance of the workshop, the video should be linked to play from this slide.** |
| 10 | A Student’s Story | Allow participants to share their perceptions. There are no right answers, per se. Take care not to let this discussion consume too much time…Remember watching the video was mostly an ‘advanced organizer’ to pique participants’ interest in what follows. |
| 11 | ? | Pause |
| 12 | Planned Happenstance Learning Theory* | PHLT was developed over the past decade by John Krumboltz and colleagues to help students manage their career trajectories. Broadly speaking, PHLT focuses on attitude and skills. |
| 13 | Attitudes of PHLT | Briefly describe each attitude and then see if participants have questions. One way to trigger conversation may be to ask, “Do any of these surprise you? Why?” |
| 14 | Skills of PHLT | Briefly describe each skill and then see if participants have questions. One way to trigger conversation may be to ask, “Do any of these surprise you? Why?” Explain that the relevance of each skill will vary across different circumstances. |
| 15 | Planned happenstance in pop culture | This slide is optional. Using pop culture can be a good way to illustrate the attitudes and skills of planned happenstance. In shorter workshops there may not be time. The reflection worksheet includes bullet points on side one for both of these stories.  Elle Woods (Reese Witherspoon) has it all. She wants nothing more than to be Mrs. Warner Huntington III. But there is one thing stopping him (Matthew Davis) from proposing: She is too blond. Elle rallies all of her resources and gets into Harvard, determined to win him back..  A billionaire industrialist and genius inventor, Tony Stark (Robert Downey Jr.), is conducting weapons tests overseas, but terrorists kidnap him to force him to build a devastating weapon. Instead, he builds an armored suit and upends his captors. Returning to America, Stark refines the suit and uses it to combat crime and terrorism.  After passing out handout, take a few minutes to review the summary of Elle’s and Tony’s stories organized by PHLT attitudes and skills. |
| 16 | ? | pause |
| 17 | Stories from Funded Research | Pass out Ben’s story. Refer to side two of handout with summary of Ben’s story.  To enhance interactivity, you may want to ask participants to complete these tasks in small groups |
| 18 | ? | Now would be a good time to address any questions about the attitudes and skills of happenstance. |
| 19 | Reflecting on Your Story | Refer to handout.  Notes space for individuals to summary the role of happenstance in their own career journeys.  Give participants time to reflect and complete their section of the handout.  Pair participants and have them tell each other their stories. |
| 20 | Tell Your story | Ask participants to form into pairs or triads |
| 21 | ? | pause |
| 22 | Looking Forward | Pull participants back as a full group.  Time permitting; you can have a large group discussion about similarities and differences in the influence of happenstance in participant’s career journeys. |
| 23 | ? | pause |
| 24 | Helping Students/peers understand  and apply PHLT | Facilitate a large group discussion of these questions.  You might want to capture ideas on a flipchart. |
| 25 | ? | Wrap up workshop.  Thanks participants and ask that participants complete the evaluation. |

# 9. References

1.	Krumboltz, J.D., *The Happenstance Learning Theory.* Journal of Career Assessment, 2009. **17**(2): p. 135-154.

2.	Krumboltz, J.D., *Capitalizing on happenstance.* Journal of Employment Counseling, 2011. **48**(4): p. 156-158.
